# CASER: A semi-supervised model with multi-omics data integration prioritizes cancer-associated epigenetic regulator genes

**DOI:** 10.1371/journal.pcbi.1014253

**Published:** 2026-04-28

**Authors:** Hao Li, Chaohuan Lin, Liyu Liu, Jie Lyu, Zhen Feng

**Affiliations:** 1 Zhejiang Key Laboratory of Soft Matter Biomedical Materials, Wenzhou Institute, University of Chinese Academy of Sciences, Wenzhou, Zhejiang, China; 2 College of Information and Engineering, The First Affiliated Hospital of Wenzhou Medical University, Wenzhou, Zhejiang, China; 3 Wenzhou Key Laboratory of Biophysics, Wenzhou Institute, University of Chinese Academy of Sciences, Wenzhou, Zhejiang, China; 4 Human Phenome Institute, Fudan University, Shanghai, China; Johns Hopkins University Whiting School of Engineering, UNITED STATES OF AMERICA

## Abstract

Prioritizing a reliable list of cancer-associated epigenetic regulators (cERs) is critical for cancer diagnosis and discovery of drug targets. While various cERs have been proposed to play important roles as cancer drivers, we anticipate that further cERs can be identified through computational analyses. In this study, we introduce a semi-supervised machine-learning approach based on tri-training model, termed Cancer-ASsociated Epigenetic Regulator identification (CASER). CASER integrates a wide range of multi-omics-derived features, including mutational, genomic, epigenetic, and transcriptomic data, to prioritize cERs as well as the four functional subtypes of cERs. When evaluated against an independent gene set, CASER demonstrates superior predictive performance compared to various other supervised machine-learning and deep semi-supervised models. CASER identified novel cERs that demonstrated cancer-driving potential and essentiality for cell survival. These novel cERs were comparable to established cancer driver genes and outperformed existing approaches for cER prediction. CASER identified dozens of novel cERs, of which six candidate cERs were shown to have roles in altering cell proliferation in four cancer cell lines. Furthermore, the prioritized cERs, particularly dual-role cERs, are more associated with anti-cancer medicines, underscoring their potential as therapeutic targets in cancer. Our study can offer valuable insights of cERs for future functional studies, advancing the understanding of their role in cancer biology.

## Author summary

The chromatin architecture of any given cell is influenced and sustained by epigenetic regulators (ERs), which are defined as a cohort of proteins capable of catalyzing the addition or removal of epigenetic marks (referred to as writers or erasers, respectively), binding to existing marks (readers), or utilizing Adenosine triphosphate hydrolysis to modify the local chromatin environment. Epigenetic drugs, commonly termed “epi-drugs”, represent a promising category of therapeutic agents that target the epigenetic modifications implicated in the regulation of gene expression. Several small-molecule inhibitors that target pivotal epigenetic enzymes, such as DNA methyltransferases and histone deacetylases, have received the approval from U.S. Food and Drug Administration for cancer treatment. These agents may function by reactivating tumor suppressor genes and promoting cancer cell differentiation and apoptosis. We employed comprehensive omics datasets to a semi-supervised model and prioritize the cancer-associated ERs, demonstrating superior performance when evaluated on independent testing gene sets and functional genomic datasets. The proposed tool outperforms existing tools in explicitly prioritizing cancer-related ERs and their subtypes. CASER identified dozens of novel cancer-associated ERs, of which six candidate cancer-associated ERs were shown to have roles in altering cell proliferation in two cancer cell lines. Furthermore, cancer-associated ERs, particularly dual-role ERs, are more associated with anti-cancer medicines, highlighting their potential as cancer therapeutic targets.

## Introduction

Chromatin is composed of DNA and the nucleosome core octamer complex including two copies of the four histone proteins that can wrap DNA. Post-translational modifications (PTMs) provide binding sites for epigenetic regulators (ERs) or transcriptional machinery [1]. The most well-studied PTMs are methylation, acetylation, phosphorylation and ubiquitination [2]. They are added by epigenetic writers, removed by epigenetic erasers, or recognized and bound by epigenetic readers [3]. Epigenetic remodelers, a special subtype of ERs that can turn condensed chromatin architecture to open state, affect target gene expression [1]. In addition, DNA methylators that recognize or bind to cytosines on DNA are also a subtype of ERs [4].

The dysregulation of epigenetic regulator genes (ERGs) has been implicated in various human diseases [5], including cancer [6]. An increasing number of ERs with either gain-of-function or loss-of-function (LoF) mutations have been extensively studied in cancer [7,8], suggesting that specific ERs may act as cancer drivers during tumor development [9]. Somatic mutations in ERGs are thought to contribute to cancer progression by enhancing cellular plasticity [10,11]. Nevertheless, a substantial proportion of ERGs lacks recurrent somatic mutations. Consequently, cancer driver genes with infrequent mutations may have been overlooked by prior cancer gene prediction methodologies.

ERs can play important regulatory roles in cancer development and progression. Previously, researchers have identified YEATS2 and ENL as cancer-associated epigenetic regulators (cERs), demonstrating that the disruption of their YEATS domains can inhibit cancer cell growth and survival [12,13]. However, only a limited number of cERs have been functionally validated in cancer, largely due to the extensive molecular experiments. A systematic understanding of the functional significance of ER dysregulation in cancer development and tumor progression remains elusive. A comprehensive identification of cERs can help enhance the understanding of the regulatory roles of ERs in cancer and contribute to the development of anticancer therapies, clinical interventions, and personalized medicine. For instance, inhibitors of DNA methyltransferases and histone deacetylases have been approved for clinical use, demonstrating the therapeutic potential of targeting ERs [14]. Moreover, the development of next-generation epigenetic drugs is underway, focusing on targeting ERs with high specificity for cancer cells. These efforts include the combination of epi-drugs with other treatment modalities such as chemotherapy, radiotherapy, and immunotherapy, which have shown promise in enhancing treatment outcomes and overcoming drug resistance [15]. Targeting ERs with small-molecule inhibitors and epigenetic drugs may offer greater therapeutic efficacy in cancer treatment compared to other anticancer therapies [9,16].

Predicting cancer driver genes is a critical area of cancer genomics research, aiming at identifying genes that contribute to tumor development and progression. However, previous cancer driver gene prediction methods were not tailored to the identification of potential cERs, and few cancer-associated ERGs can be identified from these methods. Most existing cancer driver gene prediction approaches focus on distinguishing cancer driver mutations from passenger mutations [17]. In addition, such methods may fail to capture genes with low mutational frequencies. High-resolution cancer genome sequencing analyses have shown that only a small subset of epigenetic regulator genes (ERGs) exhibit recurrent mutations in cancer cohorts [18], suggesting that additional regulatory mechanisms may contribute to ERG dysregulation in cancer. CRISPR (Clustered Regularly Interspaced Short Palindromic Repeats) screening has previously been employed to identify cancer-associated genes [19] and cERs [20,21]. Unfortunately, models based exclusively on CRISPR screening data have also demonstrated limited robustness in predicting cancer driver genes [22].

Since many ERGs can be dysregulated in cancer through mechanisms beyond somatic mutations, the models relying solely on somatic mutational data may be insufficient for identifying cERs. The advent of large-scale multi-omics datasets from extensive genome and epigenome projects has facilitated the integration of diverse omics data for characterizing cERs in pan-cancer analyses [23], providing advantages over single-omics approaches. Despite these advancements, a dedicated tool capable of predicting cERs through comprehensive multi-omics integration remains unavailable. A model that incorporates orthogonal omics datasets, including genomic, transcriptomic, and epigenomic data, could enable the identification of cancer-associated ERGs that might otherwise remain undetected in single-omics analyses.

Existing non-dedicated methods [24,25] have certain limitations in identifying cER genes. Gnad *et al.* utilized omics-derived features to predict cER genes among 187 ERGs using a *p*-value combination method [25], whereas Lu *et al.* identified 225 cER genes from The Cancer Genome Atlas (TCGA) [23] cancer samples through a robust rank aggregation approach termed as FACER (Functional Atlas of Chromatin Epigenetic Regulators) [24]. These studies incorporated a limited set of features, many of which lacked strong biological relevance and failed to account for key epigenetic characteristics. This omission may constrain the effectiveness of these methods in accurately identifying cERs. In particular, these approaches did not fully leverage available genomic and epigenomic features and their derivatives, such as H3K4me3 peak length or height and gene body methylation intensity in cancer [22,26,27], which have been proposed to play alternative roles in cancer distinct from genetic mutations [22]. Additionally, these models were unable to differentiate among specific cER subtypes, including epigenetic readers, writers, erasers, and remodelers, which are known to have distinct functional mechanisms in cancer development.

**Fig 1:**
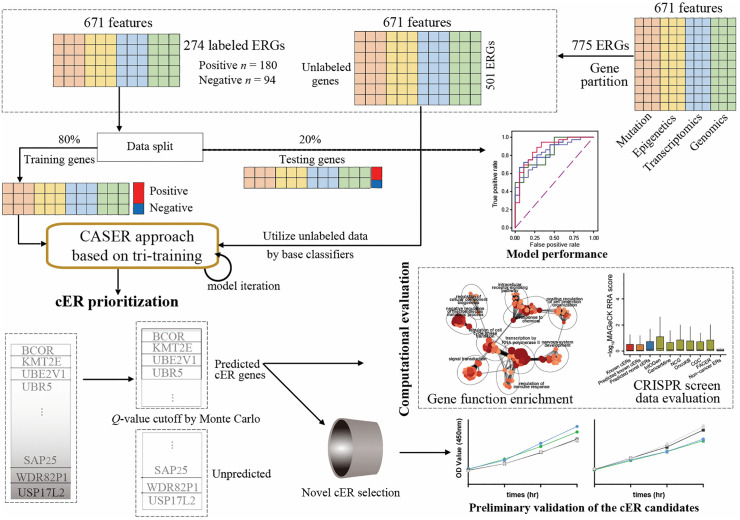
Schematic diagram of this study.

As machine-learning technology advances rapidly and with the increasing availability of large-scale multi-omics data, machine-learning-based approaches remain well-suited for identifying cancer driver genes [22], and may also be employed to identify and prioritize cER genes. Machine-learning is particularly effective for identifying cER genes with poorly characterized domains or lowly mutational frequencies, which are challenging to predict using traditional models. Machine-learning approaches that integrate multi-omics data can generate low-dimensional representations of genes from various biological features, enabling the efficient differentiation of cancer-related genes from non-cancer genes. In this study, we evaluate several machine-learning models, particularly semi-supervised models, and propose the Cancer-ASsociated Epigenetic Regulator Identification (CASER) approach, a tri-training-based model that integrates diverse multi-omics features to prioritize pan-cancer and cancer-specific cERs (**Fig 1**). CASER prioritizes cERs based on their likelihood of involvement in cancer development, aiming to identify the cancer-related cERs that have not yet been confirmed in the literature or were undetected in previous studies. We compared the prioritized cERs with known cancer genes and cER genes based on publicly available functional genomic datasets. We successfully validated six potential cERs using functional experiments across four cancer cell lines, demonstrating the potential utility of the CASER approach in identifying cERs with roles in cancer proliferation perturbation. Additionally, we also analyzed the cERs with dual roles (i.e., possessing at least two regulatory functions from readers, writers, erasers, and remodelers) or single role in gene-medicine networks. This analysis can provide novel insights into the potential value of prioritizing cERs for epigenetic drug target screening. Overall, the prediction and characterization of cERs can deepen the understanding of ERGs in tumorigenesis and support the development of novel targeted therapies.

## Results

### The evaluation of the prediction performance of several machine-learning models for the prioritization of cER genes

ERGs are characterized by their encoded proteins, which possess specific epigenetic domains (e.g., bromodomain) that endow the genes with distinct epigenetically regulatory functions. These genes can be further categorized as epigenetic remodelers or as readers, writers, or erasers of DNA or histone markers. Due to the limited number of well-characterized cERs, we aimed to identify a greater number of ERGs associated with cancer than currently recognized through developing a data-driven method, particularly based on the well-established machine-learning models. In this study, we prioritized cERs from the compiled pool of ERGs using 11 machine learning models, leveraging 671 features derived from multi-omics data, including genomic, mutational, transcriptomic, and epigenetic information, which may be relevant to cancer and/or chromatin biology (**S1 Table**). We employed all of the calculated features to train CASER, as we did not find feature selection influence the model performance. The criteria for defining positive and negative training genes are detailed in the “Training and Testing cERs” section (**S2 Table**). The independent gene sets comprised 72 cERs and 37 non-cancer ER genes (**S2 Table**) and the processed data were available in **S3 Table**.

We were initially uncertain about which machine-learning model would be more suitable for the cER prediction. Consequently, we evaluated different machine-learning models, encompassing supervised, semi-supervised (such as co-training and tri-training), and deep semi-supervised models regarding predictive performance. We specifically tested many semi-supervised models because, given the limited number of training genes, these models might outperform supervised models.

Altogether, we compared the prediction performance of 11 classification models, including seven supervised machine-learning models including Random forests (RF), support vector machines (SVM), and eXtreme Gradient Boosting (XGBoost), four semi-supervised classification models including tri-training, Assemble, co-training, and Transductive SVM (TSVM), as well as four deep semi-supervised models including FlexMatch, Unsupervised Data Augmentation (UDA), LadderNetwork, and MixMatch. We used ten-fold cross-validation (CV) and calculated the area under the precision-recall curve (AUPRC), the area under the receiver operating characteristic curve (AUROC), accuracy, F1 score, precision, and recall as the measures to compare these different models. Notably, the SVM model achieved the better accuracy, F1 score, precision and recall (**Table 1**). Therefore, we constructed the tri-training model using three SVMs as base classifiers. We systematically evaluated the different combinations of SVM kernels, specifically the Radial Basis Function (RBF), Sigmoid, and Polynomial kernels. The evaluation on the independent testing set demonstrated that the tri-training framework achieved optimal predictive performance with an ensemble comprising one RBF-kernel SVM and two Sigmoid-kernel SVMs (**S4 Table**). Utilizing this optimized configuration, the tri-training model outperformed alternative approaches, yielding superior AUROC and AUPRC scores (**Table 1**).

**Table 1:** Performance evaluation of different machine-learning models by ten-fold cross-validation.

Model	Category	Accuracy	AUROC	AUPRC	F1	Precision	Recall
SVM	Supervised learning	0.809 ± 0.083	0.852 ± 0.070	0.888 ± 0.085	0.845 ± 0.080	0.858 ± 0.107	0.837 ± 0.074
RF	Supervised learning	0.750 ± 0.089	0.829 ± 0.073	0.914 ± 0.049	0.799 ± 0.081	0.827 ± 0.108	0.779 ± 0.084
XGBoost	Supervised learning	0.768 ± 0.069	0.828 ± 0.084	0.912 ± 0.050	0.815 ± 0.066	0.834 ± 0.090	0.804 ± 0.077
TSVM	Semi-supervised learning	0.776 ± 0.031	0.816 ± 0.048	0.887 ± 0.043	0.737 ± 0.040	0.754 ± 0.040	0.731 ± 0.040
Co-training	Semi-supervised learning	0.727 ± 0.113	0.815 ± 0.082	0.897 ± 0.063	0.656 ± 0.115	0.735 ± 0.126	0.659 ± 0.095
Tri-training	Semi-supervised learning	0.814 ± 0.080	0.882 ± 0.088	0.924 ± 0.060	0.786 ± 0.084	0.791 ± 0.084	0.723 ± 0.087
Assemble	Semi-supervised learning	0.459 ± 0.107	0.828 ± 0.065	0.906 ± 0.067	0.424 ± 0.116	0.614 ± 0.154	0.586 ± 0.065
FlexMatch	Deep semi-supervised learning	0.668 ± 0.091	0.564 ± 0.084	0.690 ± 0.128	0.476 ± 0.103	0.501 ± 0.204	0.540 ± 0.049
UDA	Deep semi-supervised learning	0.686 ± 0.121	0.666 ± 0.132	0.797 ± 0.091	0.516 ± 0.154	0.538 ± 0.104	0.583 ± 0.104
LadderNetwork	Deep semi-supervised learning	0.745 ± 0.042	0.867 ± 0.042	0.928 ± 0.043	0.733 ± 0.054	0.751 ± 0.058	0.783 ± 0.044
MixMatch	Deep semi-supervised learning	0.714 ± 0.095	0.754 ± 0.095	0.844 ± 0.103	0.592 ± 0.129	0.655 ± 0.157	0.622 ± 0.091

Values are represented by mean and standard deviation.

**Fig 2:**
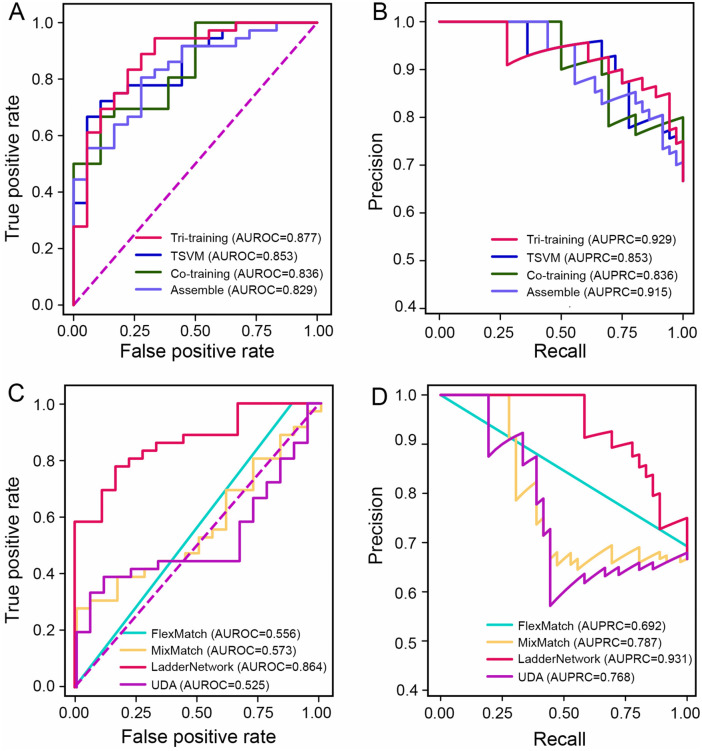
Performance of different semi-supervised models based on the independent testing gene set. **(A)** The area under the receiver operating characteristic curve (AUROC) for four semi-supervised models. **(B)** The area under the precision-recall curve (AUPRC) for four semi-supervised models. **(C)** The AUROC for four deep semi-supervised-learning models. **(D)** The AUPRC for four deep semi-supervised-learning models. The FlexMatch model fails to capture patterns in the omic data, producing predictions no better than random chance, a consequence of fundamental failures in its pseudo-labeling mechanism.

We then tested all of the models based on an independent set of testing genes. Our evaluation indicated that semi-supervised models, such as co-training and tri-training, outperformed other models based on the highest values in different metrics (**Figs 2** and **S1**). Similarly, we also observed in **Fig 2** that the tri-training model outperformed other models (AUROC = 0.877 and AUPRC = 0.929). The model performance of all of the semi-supervised models was comparable for CV and independent testing, suggesting that no apparent overfitting was found for semi-supervised models (**Table 1** and **Fig 2**). The AUROC values of the semi-supervised models were generally higher than other models regarding the cER prediction performance (**Fig 2**). A deep semi-supervised model, LadderNetwork, also achieved comparable performance with tri-training model (**Table 1** and **Fig 2**). Regardless, tri-training was chosen in the following model building as its better interpretability, shorter running time, and better performance. We termed our tri-training-based model as CASER and applied it to prioritize cER genes. When training CASER on randomly selected subsets comprising 40%, 60%, and 80% of the original training data, we noted a marginal decrease in predictive performance on the independent test set, suggesting the robustness of CASER (**S2 Fig**). Detailed information regarding the CASER model is presented in **Fig 1** and **S3 Fig**. We additionally evaluated three state-of-the-art deep-learning architectures, including Graph Attention Network (GAT), Graph Convolutional Networks (GCN), and GraphSAGE. However, all exhibited significantly lower performance than CASER based on the independent testing set. (**S1****C-****S1D Fig**).

We examined the contribution of distinct non-overlapping feature subsets to the prediction on the independent testing set, thereby enhancing its transparency. For this purpose, we constructed various CASER model variants based on the following feature subsets: ‘Mutation’, ‘Genomics’, ‘Transcriptomics’, ‘Epigenetics’. Subsequently, we compared the AUPRC and AUPRC for these model variants, as illustrated in **S4****A** and **S4B Fig**. Our analysis revealed that these unique feature categories may be complementary, underscoring the significance of integrating a comprehensive set of features (**S4****A** and **S4B Fig**). Notably, epigenetic and mutational features exhibited a greater contribution to the model performance (**S4****A** and **S4B Fig**), indicating that epigenetic dysregulation might also play a pivotal role in the genomic-scale dysregulation of ERGs in cancer. We further quantified feature importance by computing the mean absolute SHapley Additive exPlanations (SHAP) values for each input, thereby identifying the features that most strongly influence CASER predictions (**S5 Fig**).

### The prediction of cERs reveals many novel cER genes implicated in cancer

CASER has demonstrated great efficacy as a predictive model for cERs in the evaluation of model performance. Consequently, we employed CASER to predict pan-cancer cERs as a whole and across four distinct subtypes: epigenetic reader, writer, eraser, and remodeler. The parameters utilized in the models for the four cER subtypes were consistent with those of the overall cER prediction model.

**Fig 3:**
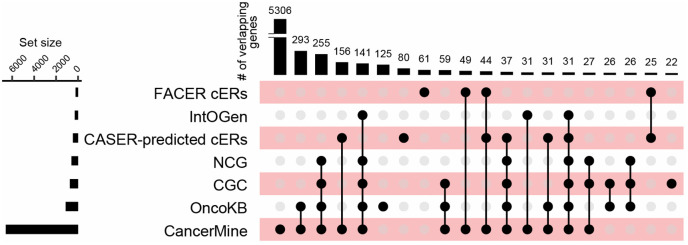
UpSet plot showing the overlap between the identified cER genes with the cancer genes from different databases. Upset plot diagram shows the intersection of the six cancer gene sets and the identified cER genes. Vertical bars (indicated by the black dots joined by black lines) represent the number of genes in the different combinations of cancer gene sets. Horizontal bars represent the number of genes in the different cancer gene sets.

CASER assigned each ERG a cER score ranging from 0 to 1, reflecting the probability of the ERG being associated with cancer. A higher cER score denotes an increased likelihood of the gene being classified as a cER gene. Each cER score generated by CASER was accompanied by a measure of statistical significance. Utilizing a *q*-value threshold of 0.1, a total of 460 ERGs were identified as potential cER genes, including 300 novel cER genes (**S5 Table**). Novel cER genes were identified as those predicted cERs that were not in the training or testing gene sets. The predicted novel cERs, along with subtype-specific novel cERs, are detailed in **S6 Table**. Many of these predicted novel cERs have been underexplored in previous research, and their potential role in epigenetic mechanisms related to cancer remains unclear. We assessed the extent to which these cERs have been documented in established cancer driver databases, such as Cancer Gene Census (CGC) [28], CancerMine [29], Network of Cancer Genes (NCG) [30], IntOGen [31] and OncoKB [32], as well as among the 225 FACER-predicted cERs. Our findings revealed that a significant number of cERs were indeed already known as cancer driver genes. Notably, 80 cERs were novel cancer genes, not previously catalogued in these prominent cancer driver databases (**Fig 3** and **S7 Table**). At least 50 of these genes have been documented in the literature as cancer epigenetic drivers (epi-drivers) or cancer-related genes (**S7 Table**), while an additional 30 genes warrant further experimental validation and functional analysis. Moreover, many novel cERs, which were not identified by previous cancer driver prediction tools, demonstrated superior rankings compared to training cERs when assessed by CASER. These cER genes may also be cancer driver genes with non-canonical mutational patterns, as traditionally recognized cancer genes were typically identified by their high mutational frequency. For example, *ZMYND11* gene, identified as one of the cERs (*q*-value = 0.0) and cancer-related readers (*q*-value < 0.1) by CASER, was not included in the training or testing gene sets. A recent finding indicated that *ZMYND11* functioned as an ER reader and exhibited a tumor-suppressive role in cancer through its tandem bromo-PWWP domains [33], which was consistent with the CASER prediction and cancer-associated epigenetic reader prediction.

We also independently predicted four cER subtypes using the CASER model (**S6 Fig**), utilizing the training gene sets specified in **S2 Table**. Our analysis identified 133, 112, 45, and 39 cERs as cancer-associated epigenetic readers, writers, erasers, and remodelers, respectively (**S5 Table**). Notably, 67 reader, 56 writer, 19 eraser, and 14 remodeler cERs were not present in the subtype-specific training gene sets (**S5 Table**). Furthermore, the subtype-specific cER prediction results enabled us to obtain the cER genes with potential dual-functional roles across the categories of epigenetic readers, writers, erasers, and remodelers, a benefit of the independent prediction of the four ER subtypes using the CASER model.

**Fig 4:**
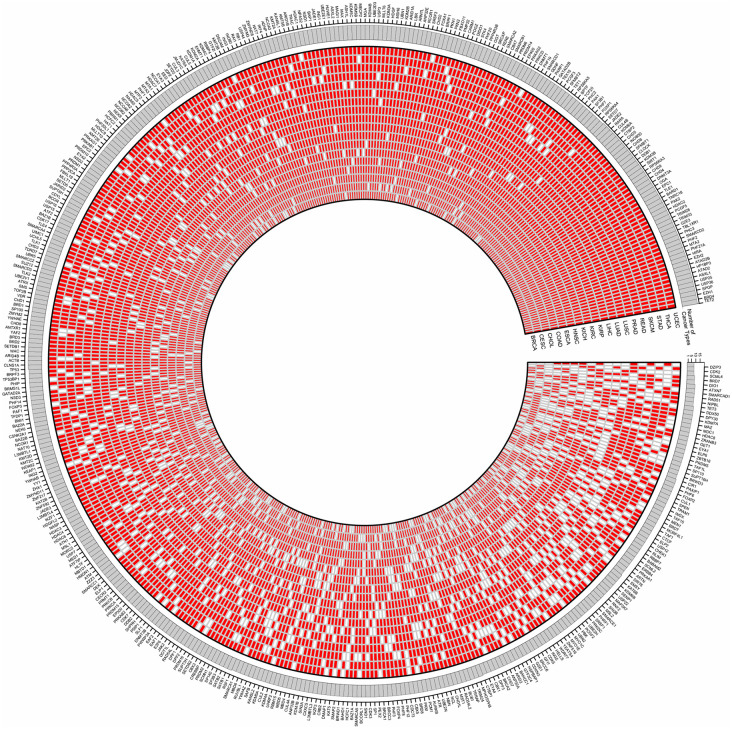
The Circos plot displays the predicted cancer-specific cER genes across 18 cancer types. The predicted cER genes are highlighted in red, and the outermost ring contains the corresponding gene symbols.

The dual-role cER gene prediction was obtained by summarizing the four subtype-specific models, which achieved an excellent CV performance (Accuracy (confident interval) = 0.8538 (0.7812, 0.9097)). We found 32 novel dual-role cER genes (*ASH1L*, *ASXL1*, *ATAD2*, *ATRX*, *BAZ1B*, *BAZ2A*, *CHD1*, *CHD2*, *CHD3*, *CHD4*, *CHD5*, *CHD8*, *CHD9*, *CREBBP*, *DNMT1*, *EHMT1*, *EP300*, *KAT6A*, *KAT6B*, *KDM2A*, *KDM2B*, *KDM4A*, *KDM4B*, *KDM4C*, *KMT2A*, *KMT2B*, *KMT2D*, *KMT2E*, *RAI1*, *SMARCA4*, *SUV39H2, TAF1*) in addition to 16 known dual-role cER genes that were already implicated in cancer, suggesting that many previously uncovered dual-role cER genes may have dual roles in cancer epigenetic regulation. For instance, *PHF6* (PHD finger protein 6), a gene previously classified as an epigenetic reader, was predicted as an epigenetic remodeler in cancer, a finding corroborated by a recent study [34]. Furthermore, *CHD8* (chromodomain helicase DNA binding protein 8), which lacks domain annotation in the pFAM database [35], is predicted by CASER to serve as a dual-role cER gene, encompassing both reader and remodeler functions, aligning with the findings from another prior study [36]. We also identified cER genes separately for each of the 18 common cancer types in TCGA. The prediction results were shown in **Fig 4** and the details can be found in **S8 Table**.

In summary, the subtype-specific predictions of cER genes offer the research community a valuable repository of ERGs that may function differently in context-specific epigenetic regulation. In the subsequent sections, we focused on exploring the prioritized cERs through comprehensive bioinformatic and experimental assessments. However, we did not conduct these analyses for the predicted subtype-specific cERs, with the exception of gene network analysis, due to the insufficient number of genes within specific subtypes to satisfy the statistical requirements necessary for bioinformatic analysis.

### Characterization of CASER-prioritized novel cERs by functional genomic datasets shows further evidence of their association with cancer

The functional genomic datasets that were generated from large-scale omics technologies enabled the interrogation of the prioritized novel cER genes. Several functional genomic datasets were employed to evaluate the cancer relevance of the novel CASER-predicted cER genes, in comparison with established cER genes and non-cancer genes (NGs). We also used the unpredicted ERs (*q*-value > 0.1) as an alternative negative control gene set.

**Fig 5:**
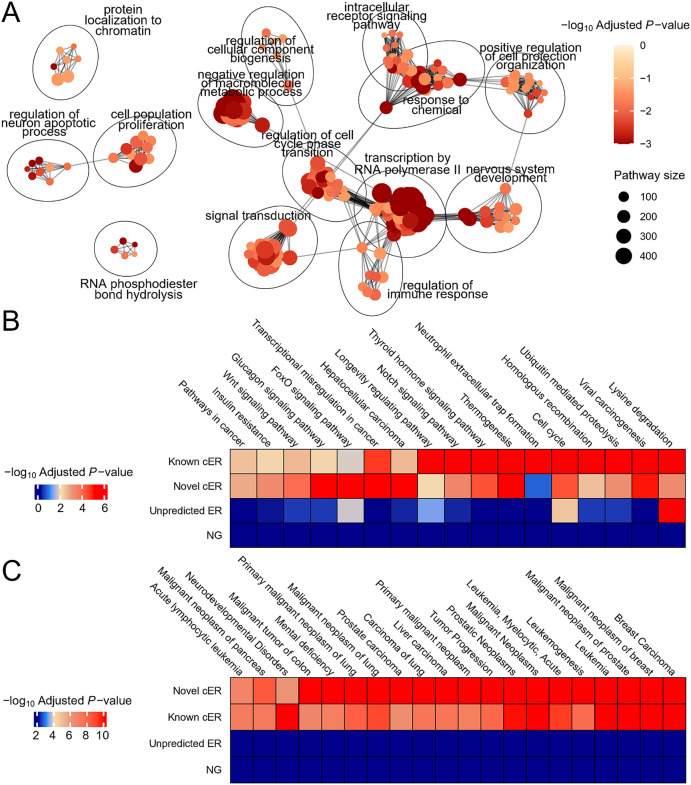
Evaluation of CASER-predicted cERs by gene set enrichment analysis. **(A)** Network visualization of the enriched pathways based on the gene set enrichment analysis results of GOBP pathways. Node represents a specific GOBP pathway, and edges represents numbers of shared genes. The size of nodes corresponds to the number of genes in that pathway, while the color gradient represents the -log_10_ adjusted *p*-value, with darker red colors indicating higher pathway enrichment. **(B)** KEGG pathway and **(C)** DisGeNET gene set enrichment results for the predicted cERs (including known and predicted novel cERs), unpredicted cERs and NGs. Terms with adjusted *P*-values < 0.01 are shown.

Firstly, we conducted a Gene set enrichment analysis (GSEA) [37] on the predicted cERs with a *q*-value threshold of less than 0.1, utilizing the Gene Ontology [38] Biological Process (GOBP) gene set through the aPEAR R package [39]. The enrichment analysis revealed that the majority of the aggregated GOBP terms were significantly associated with tumorigenesis, such as ‘intracellular receptor signaling pathway’, ‘regulation of cell cycle phase transition’, ‘cell population proliferation’, ‘positive regulation of cell projection organization’, and ‘regulation of immune response’ (**Fig 5A**).

The Kyoto Encyclopedia of Genes and Genomes (KEGG) [40] pathway enrichment analysis that was performed on both predicted and known cERs revealed a significant enrichment of KEGG terms closely associated with tumorigenesis, such as ‘Pathways in cancer’ and ‘Cell cycle’ (**Fig 5B**). Additionally, other enriched pathways align with the established roles of cancer-related genes in cancer cellular biology. Conversely, the KEGG pathway analysis of unpredicted ERs (those not classified as cERs) and NGs (a negative control) did not exhibit notable enrichment in most of these pathways (**Fig 5B**). Furthermore, the enrichment tests on DisGeNET (Gene-disease networks) gene sets [41] were also performed to explore the association of these gene sets with human diseases. Notably, the majority of enriched DisGeNET terms for the predicted and known cERs were related to cancer biology (**Fig 5C**). In contrast, no terms were enriched for the unpredicted ERs and NGs (**Fig 5C**).

We also used a published Assay for Transposase-Accessible Chromatin with high-throughput sequencing (ATAC-seq) dataset comprising dozens of TCGA pan-cancer samples to characterize the FACER-predicted novel cancer driver genes [42]. Based on this ATAC-seq dataset, we found that FACER-predicted novel cERs and known cERs are significantly more accessible than unpredicted ERs and non-cancer ERs in pan-cancer samples and cancer-specific samples (*P*-values < 0.05 by the two-sided Wilcoxon rank-sum test) (**S7 Fig**). This result suggested that the predicted cERs are ubiquitously accessible in cancer samples.

Then, we assessed the clinical relevance of the cERs predicted by CASER. In particular, we analyzed the predicted novel cancer-specific cER genes alongside other gene sets using the cancer patient survival data from the patients. We utilized the hazard ratio (HR) data, which was precomputed by the OncoRank platform (http://www.oncolnc.org). An HR greater than 0 suggests that elevated expression of a specific cER is associated with reduced survival time in cancer patients, whereas an HR less than 0 implies that increased expression of a specific cER is linked to prolonged survival time. We conducted a comprehensive analysis across all TCGA cancer types for which survival data were available and determined that CASER-predicted cERs, encompassing both novel and known cERs, may serve as risk factors in Glioblastoma multiforme (GBM), Esophageal carcinoma (ESCA), and Acute Myeloid Leukemia (AML), while acting as protective factors in other cancer types (**S8 Fig**). The results suggested that the role of cER genes in cancer may differ across various cancer types.

**Fig 6:**
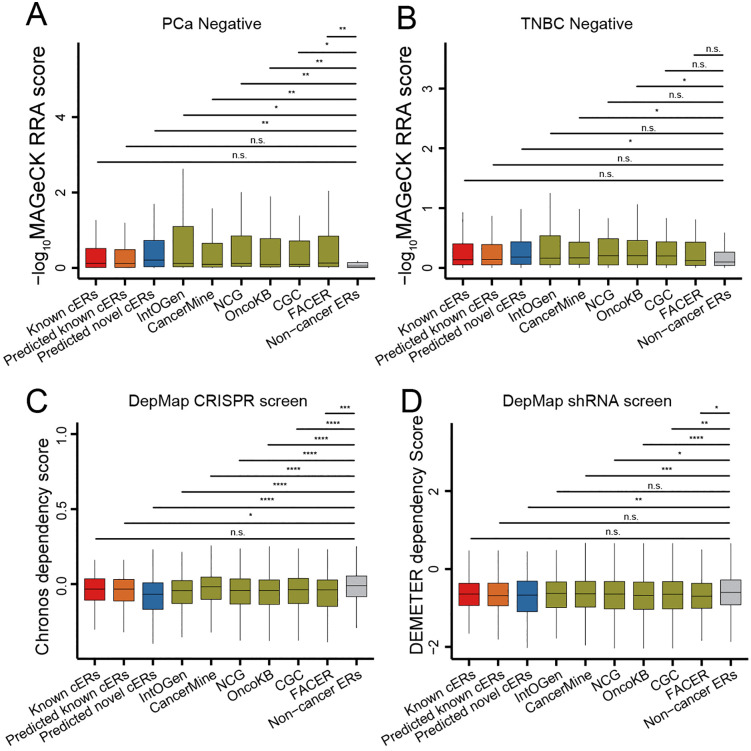
Evaluation of the identified cERs by a published ER CRISPR-screen dataset and the DepMap gene essentiality screen dataset. The robust rank aggregation (RRA) scores of negative selections are shown for different gene sets in **(A)** LNCaP and **(B)** MDA-MB-231 cells. The Chronos dependency scores are shown for different gene sets in **(C)** LNCaP and **(D)** TNBC cancer cell. The RRA scores of negative selection were calculated using MAGeCK. The higher -log_10_(RRA) values indicate a greater effect on cell survival after gene knockdown. The lower DEMETER and Chronos score indicate a greater effect on cell survival after gene knockdown. The genes in this analysis are both cancer genes and ERGs. Non-cancer ERs refer to the ER genes that are also neutral genes. *P*-value is calculated by Wilcoxon rank-sum one-tailed test.

To enhance the understanding of the role of CASER-identified cERs in cancer cell survival, we conducted a benchmarking analysis comparing CASER-identified cERs with various gene sets, including different cancer driver gene lists, a non-cancer driver gene list, and a cER gene list predicted by the previous study FACER [24], utilizing available CRISPR screen data. Firstly, we utilized a previously published CRISPR dataset generated by Ozlem *et al.* [20], which employed a CRISPR-Cas9 knockout library (EPIKOL) to assess the impact of cER gene knockout on overall cell survival. Our analysis revealed that the predicted cERs exerted a comparable influence on the survival of two cancer cell lines, in conjunction with established cancer genes and FACER-predicted cERs. Notably, the predicted cERs by CASER and FACER and the most of the cancer gene sets (keeping only ERGs) demonstrated a significantly greater effect on cancer cell survival compared to the NG controls (**Fig 6A** and **6B**).

Subsequently, we conducted a similar evaluation of the CASER-predicted novel cER genes utilizing the publicly accessible pan-cancer CRISPR and shRNA screening datasets from the dependency map (DepMap) project [43]. The dependency scores, calculated by Chronos [44], which accounts for batch effects and off-target influences, offered more precise evaluations of functional impact compared to Model-based Analysis of Genome-wide CRISPR/Cas9 Knockout (MAGeCK) [45] across diverse experimental settings. A Chronos score of zero signifies no effect on cell viability, while a negative dependency score denotes a reduction in cell viability resulting from gene knockdown. The lower the Chronos score, or its predecessor DEMETER [43] score, the more crucial the gene is for cell survival. The analysis revealed that the knockdown of CASER-predicted novel cER genes exhibited gene essentiality scores comparable to those of known cERs and cancer driver genes (keeping only ERGs) (**Fig 6C** and **6D**). Furthermore, these scores were significantly more negative than those of NGs. The CASER-predicted novel cERs demonstrated a negative impact on cancer cell viability upon knockdown, akin to known cERs and cancer driver genes (**Fig 6C** and **6D**). The predicted cERs, particularly those identified by CASER, along with cancer driver genes, demonstrated a significantly negative impact on cancer cell survival when compared to NGs in CRISPR screen data (**Fig 6C**). In the context of shRNA screen data, this trend persisted, albeit with a minor difference (**Fig 6D**). Taken together, the gene essentiality of CASER-predicted novel cERs was generally comparable to that of genes identified by the previous FACER study [24], and even marginally superior to FACER-predicted cERs in certain assessments. Furthermore, CASER significantly expands the cER gene repertoire by providing alternative cER candidates for subsequent experimental validation.

### Preliminary validation of the predicted cER genes in cancer cell lines

**Fig 7:**
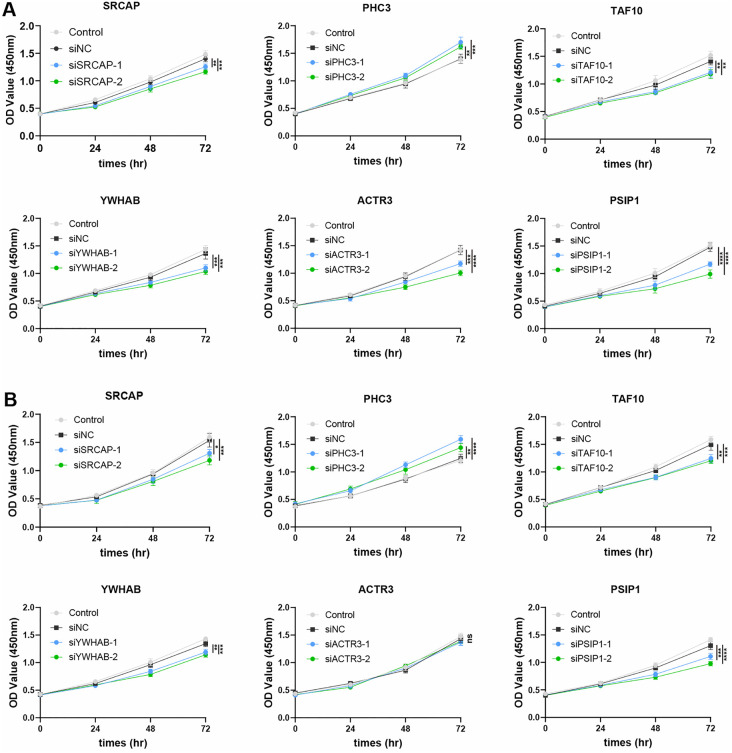
Investigation of six candidate cER genes in affecting the proliferation of cancer cell lines. **(A)** Six predicted cER genes were investigated in SK-mel-2 cell line (*n* = 4). **(B)** Six predicted cER genes were investigated in Caki-1 cell line (*n* = 4). Statistical analysis was performed between si-NC and si-Genes. *P*-values are calculated by One-way analysis of variance followed by Dunnett’s corrections and indicated by star symbols, *, *P* < 0.05; **, *P* < 0.01; ***, *P* < 0.001; ****, *P* < 0.0001; ns, *P* > 0.05.

To validate the CASER model as a proof-of-concept, we selected six predicted cER genes to test their roles in cancer proliferation, including *PHC3* (polyhomeotic homolog 3), *SRCAP* (Snf2 related CREBBP activator protein), *TAF10* (TATA-box binding protein associated factor 10), *YWHAB* (tyrosine 3-monooxygenase/tryptophan 5-monooxygenase activation protein beta), *PSIP1* (PC4 and SRSF1 interacting protein 1), and *ACTR3* (Actin Related Protein 3). These genes were identified as cERs by CASER in four cancer cell lines except *ACTR3*. *ACTR3* was only predicted as cancer driver genes in Skin Cutaneous Melanoma (SKCM) and Prostate adenocarcinoma (PRAD). These genes were investigated in four cancer cell lines which are representative of four common cancer types. A comprehensive review of the literature regarding these genes in the context of cancer suggests that the evidence supporting their involvement in cancer remains inconclusive, warranting further investigation into their roles in common cancer cell proliferation. Given the high expression levels of these genes in the four cancer cell lines in the DepMap gene expression data, we employed two distinct siRNAs to silence these genes in cell lines. The results demonstrated that the siRNA treatment reduced the mRNA expression by at least 30%, after 48 hr of transfection (**S9 Fig**). In the four cell lines, the downregulation of these genes via siRNAs resulted in a significant reduction or increase in cell proliferation rates, as evidenced by Cell Counting Kit-8 (CCK-8) assays, when compared to negative control transfections (**Figs 7 and** **S10**), suggesting a potential role for these genes in carcinogenesis. Available cell proliferation results for *ACTR3* and *YWHAB* in other cell lines are also consistent with our findings [46,47]. Taken together, these genes were involved in cancer cell proliferation, which can be further investigated in future research to explore the underlying mechanisms.

### Characterization of cERs in gene-medicine network

Cancer genes are important targets of anti-cancer drugs and personalized cancer therapies. Consequently, we assessed the associations between the predicted cERs and anti-cancer medicines based on a pharmacogenomic dataset, PharmacoDB [48]. PharmacoDB contains a large number of interactions between genes and anti-cancer drugs, thereby requiring network module analysis to help a better comprehension of their relationships (**S11 Fig**). To minimize potential side effects of medicines, we have utilized only the gene-drug relationships with an adjusted *p*-value < 0.01 from the drug sensitivity analysis provided by the PharmacoDB database in this study. Our analysis revealed that dual-role cER genes exhibited higher node degrees compared to single-role cER genes, non-predicted ERGs, and NGs (**S12 Fig**). The Molecular Complex Detection (MCODE) algorithm [49] was applied to identify densely connected network modules for the predicted cERs within the gene-medicine network. Within the identified modules (**S11B Fig**), 7 out of 11 modules comprised dual-role known or novel cERs. The dual-role cERs were predominantly found in densely connected modules. The enriched dual-role cERs within the modules exhibited a significantly higher degree of overrepresentation (*P*-value = 0.004, one-tailed binomial test) compared to single-role cERs (*P*-value = 0.051) and non-predicted ERs (*P*-value = 0.110). This finding suggested a greater potential for the prioritized cERs to serve as medicine targets for cancer therapy.

## Discussion

A substantial number of ERGs are crucial in the initiation and progression of cancer. Historically, researchers in the field of cancer epigenetics have predominantly relied on the cancer genes documented in existing literature or public databases as a foundation for conducting subsequent experiments, which may introduce researcher biases. The identification of novel epi-driver genes using computational tools has generated great interest within this field recently. In this study, we introduce a machine-learning-based approach, CASER, designed to prioritize cER genes (**Fig 1**). The primary objective of this study is to transform a list of ERGs into a ranked list of cER genes and to identify the most reliable candidates for subsequent functional characterization. Furthermore, we aim to independently prioritize the four subtypes of cERs, an aspect that has not been systematically addressed in previous studies. To this end, CASER integrates a diverse array of features derived from multi-omics data to predict cERs. The unprecedented scale of omics data, coupled with numerous data processing tools, presents a valuable opportunity to expand the authentic list of cER genes. CASER not only successfully recovers known cERs but also identifies novel cER genes that have not been previously documented in the literature. The characterization of the CASER-predicted cERs shows that it outperforms the previous cER identification method FACER. While the oncogenic role of the six candidate cERs in cancer requires further extensive investigation, current evidence already indicates the cancer-driving potential of the validated cERs, highlighting the usefulness and reliability of CASER. The dozens of novel cERs, which are not documented in the literature, may aid in the identification of new therapeutic targets for cancer. However, CASER could not pinpoint the specific loci driving cancer development, as its predictions are gene-centric. A recent method, termed Mutations of ERs in Perturbed Interactions (MERIN) [11], may serve as a promising approach for predicting cancer-related epigenetic domains in ER genes. Future studies can further identify the cancer driver mutations of the predicted cERs, which can be the ideal drug targets for cancer therapy.

Semi-supervised learning has gained prominence as a robust alternative to conventional supervised learning, especially in contexts where the number of known cER genes is limited. By leveraging both data of known cER genes and uncertain genes, semi-supervised learning can potentially improve the accuracy of cER predictions. Notably, the tri-training models employing SVMs as base classifiers demonstrated superior predictive performance compared to traditional supervised models and several other semi-supervised models (**Table 1** and **Figs 2** and **S1**). In tri-training frameworks, parameters are derived not only from labeled data but also from a substantial volume of unlabeled data, facilitating a more comprehensive capture of the underlying data distribution, thereby diminishing the dependency on known cERs and potentially reducing the costs associated with cER annotation. This approach contrasts with supervised learning models, which are prone to overfitting when trained on limited labeled data and consequently exhibit poor generalization to unseen data [50]. Consistently, the downsampling of the training data only marginally decrease the performance of CASER (**S2 Fig**). The result demonstrates that CASER does not heavily rely on the full scale of the training data to learn effective decision boundaries, highlighting the model’s strong generalization capabilities even when trained with limited training genes. Furthermore, tri-training operates by iteratively selecting the most confident predictions from each of the three basis classifiers, which can help reduce the risk of overfitting and enhance the robustness of the model. Compared to other approaches, tri-training offers the advantage of using diverse base models, making it particularly well-suited for situations where the labeled data is scarce but a large pool of unlabeled data is available. Moreover, tri-training has been shown to work well in modeling omics data, which can be noisy and incomplete. By using multiple classifiers, each trained on different subsets of the data, the approach allows for a greater utilization of available information, which can improve the predictive performance when labeled data is limited. Based on individual model performance (**Table 1**), we selected SVMs as the base classifiers for our tri-training model, because exhaustively screening diverse base models is computationally prohibitive. Our preliminary evaluation to highly efficient classifiers (KNN, linear regression, XGBoost, and SVMs) suggested that the proposed SVM-centric configuration outperformed these alternatives on the testing data. Additionally, because kernel selection heavily influenced predictive accuracy (**S4 Table**), we integrated SVM classifiers using one RBF and two sigmoid kernels into CASER, which maximized the performance on the independent test set. Overall, tri-training provides significant advantages over supervised learning by effectively leveraging unlabeled data to enhance model performance and improve generalization. The LadderNetwork model, integrating supervised and unsupervised learning through a unique encoder-decoder structure with lateral skip connections, also shows excellent prediction performance (**S1 Fig**). In principle, LadderNetwork allows the model to simultaneously optimize classification performance and reconstruction accuracy, making it an alternative model for cER prediction. Given the limited scope of the models we have tested, the predictive performance of cER genes could potentially be further improved by employing more deep-learning models [51], though three deep-learning models were tested (**S1****C-****S1D Fig**).

Previous studies have characterized ERGs primarily based on canonical features such as mutational patterns [9,25]. However, cER genes that undergo somatic mutations and/or focal amplification/deletion in cancer do not constitute the majority of the entire ERG repertoire. Notably, epigenetic information, which is independent of somatic mutations and genomic features, may provide orthogonal insights that facilitate the prioritization of novel cER genes that may be challenging to identify using genomic data alone. Our hypothesis is informed by prior research indicating that specific epigenetic patterns are linked to cancer driver genes [26,27]. Significantly, the CASER variant, which incorporates only epigenetic features, accounts for the majority of the predictive performance (**S4 Fig**). This suggests that ERGs may be modulated by epigenetic regulation from other ERs. Further investigation into the interactions and mutual influences among ERGs could be valuable in the context of cancer drug development, as unexpected ERG interactions might impact the effectiveness and safety of targeted cancer inhibitors. Considering the interplay among certain ERs in the coordinated regulation of tumor progression, the simultaneous application of two or more inhibitors targeting different ERs is likely to yield more favorable outcomes in overcoming drug resistance [52].

The independent prioritization of different subtypes of cERs significantly enhances the dual-role cER gene reservoir, representing a notable advantage of the proposed CASER model. To the best of our knowledge, there is currently no bioinformatic characterization comparing dual-role cER genes to single-role cER genes from previous studies. Although the specific predicted dual-role cERs remain unexplored in detail, we anticipate that future biochemical studies will deepen our understanding of the multifaceted roles these cERs play in cancer regulation. Notably, we identified a distinctive characteristic of dual-role cERs: the dual-role cER genes exhibit increased connectivity within the gene-medicine network (**S12 Fig**), suggesting that cERs, particularly dual-role cERs, may serve as promising drug targets. This conclusion holds significant practical implications for pharmacological interventions in cancer. However, it is important to note that this conclusion is based solely on our large-scale analyses and does not necessarily imply a direct or causal relationship for specific cERs. The molecular specificity of drugs, as well as their potential side effects, may contribute to false positives. Therefore, while gene-medicine networks offer valuable insights into potential therapeutic targets, their interpretation should be approached with caution. It is important to recognize that the involvement of specific medicines in cancer treatment may require further validation through additional analyses, including experimental and clinical validation. In addition, the presence of dual-role cERs introduces additional challenges to the development of anti-cancer drugs and the clinical treatment of cancer, due to the potential side effects associated with targeting an ER gene that fulfills multiple epigenetic regulatory roles. Furthermore, the dual functionality of cERs encourages a re-evaluation of well-known cER genes and the exploration of potential new roles in cancer epigenetic regulation in future research endeavors.

Our study primarily focuses on computational predictions and bioinformatics analysis to evaluate the predicted cERs, utilizing publicly available datasets such as TCGA, DepMap and other resources. While these datasets provide a valuable foundation for investigating molecular patterns and survival outcomes across diverse patient populations, they do not offer direct experimental validation. Several genes were subsequently validated through preliminary cell proliferation experiments. *In vivo* validation, such as through animal models, three-dimensional organoid cultures or patient-derived tumor samples, was not included in this study. Future research could utilize such techniques to validate the cancer driving potential of the predicted cERs, which would provide more definitive evidence of their functional roles in cancer progression and further enhance the clinical relevance of our findings.

In summary, the integration of the omics data with specific semi-supervised machine-learning models is efficient for advancing the prediction of cER genes. CASER not only improves the identification of candidate cER genes but also contributes to a more comprehensive understanding of ERGs in cancer, ultimately aiding the refinement of precision medicine strategies. This study highlights the value of incorporating omics-derived features into machine-learning models to prioritize cER genes, thereby offering mechanistic hypotheses for further investigation. Despite the utilization of an extensive array of features to enhance predictive capabilities, the continuous expansion of functional genomic and epigenomic data presents ongoing opportunities for further refinement of the prediction model. Future investigations can continue the development of more innovative machine-learning methodologies for the prediction of cER genes. This study provides novel insights into human cER genes; however, additional research is required to elucidate the regulatory mechanisms and evolutionary origins of prioritized cER genes, as well as to obtain further biological evidence regarding the positioning of cERs within molecular networks and their biological implications. Altogether, our research prioritized hundreds of cERs and found 80 novel potential epi-drivers, thereby establishing a foundational framework for chromatin biology and related disciplines, particularly in the advancement of targeted therapies and personalized medicine for cancer treatment.

## Materials and Methods

### Gene annotations

The human ERGs were derived from a previous study [53]. The functional subtypes (reader, writer, eraser, and remodeler) of ERs were obtained from the CR2Cancer database [54]. The ERG list and related annotations can be found in **S2 Table**. ENSEMBL gene annotation (version 87) was used as the genome-wide gene annotation. The gene annotations and multi-omics datasets from hg19 human genome version were downloaded. If not hg19 version, data was subject to genome version conversion to hg19 by using the LiftOver program (https://genome.ucsc.edu/cgi-bin/hgLiftOver). Gene promoters were defined as the region of upstream 1,000 base pairs (bp) to downstream 500 bp of transcription start sites (TSSs) of genes, while gene-body regions were defined as the remaining regions within TSSs and transcription termination sites.

Many ERGs may primarily be implicated in genetic or neurological disorders rather than in cancer [5]. The negative gene set used to evaluate model performance was defined based on the neutral gene set from a previous study [22], consisting of 4,202 genes, excluding known cancer driver genes.

### Multi-omics features

The selection criteria for incorporating these features into the models were grounded in established or potential evidence of their associations with cancer or epigenetic regulation. A total of 671 gene-centric features were collected to become a set of multi-omics data derived features. Features were grouped into the following classes: ‘Mutation’, ‘Genomics’, ‘Epigenetics’, and ‘Transcriptomics’. Further details regarding these features can be found in S1 Table. To mitigate potential researcher biases, the features derived from literature and manually curated knowledge bases were excluded. Furthermore, we opted not to include features related to biological networks, such as protein-protein interaction networks, in our model. Although these features have previously been demonstrated to be effective predictors of cancer genes, they tended to be biased towards well-studied genes [55]. We harmonized different datasets based on official gene symbol. The unmatched gene aliases were converted to official gene symbol.

#### Mutational features.

The somatic mutational datasets were downloaded from the data from the Catalogue Of Somatic Mutations in Cancer (COSMIC) v98 [56]. The samples that are present in more than one dataset or hypermutated with more than 2,000 mutations were excluded. The final dataset contains over eight million somatic mutations from > 30 cancer types and was used for the calculation of mutational features.

The Variant Effect Scoring Tool (VEST) scores and SNVBox features as provided by the Cancer Related Analysis of Variants Toolkit (CRAVAT) tool [57] (feature ID: 1 ~ 80) to measure the functional impact of somatic mutations were included. Many candidate mutational features were previously defined by Davoli *et al.*’s [58] and Tokheim *et al.*’s paper [55]. For example, PolyPhen2 HumVar prediction model [59] was utilized to evaluate the functional effects of missense mutations (ID: 81) and to divide them as either high functional impact (HiFI) or low functional impact (LoFI) [58] based on binary and probabilistic outputs from the PolyPhen2. In addition, the various ratio-metric features (ID: 82~106) were quantified using the script provided by Davoli *et al.* [58]. For example, we used the features derived from HiFI and LoFI, including: benign mutations (Silent and LoFI missense mutations), LoF mutations (splicing, nonsense and frameshift mutations); and HiFI missense mutations (damaging missense mutations). Splicing mutations are those affecting splicing sites. Inactivating mutations include splice site, translation start site, indel frameshift, and nonstop mutations. PolyPhen2 score was obtained from the PolyPhen-2 web server (http://genetics.bwh.harvard.edu/pph2/) [60]. In addition, the VEST pathogenicity score [61] for missense mutations were calculated by the CRAVAT online website (http://www.cravat.us/CRAVAT/)[57]. For the ratio-metric features (ID: 82 ~ 106) in **S1 Table**, a pseudocount was added to avoid division by zero. The FATHMM score (ID: 107) [62] that also measures the functional consequences of somatic mutations was also used [63]. We also used the somatic selection coefficient features (ID: 108 ~ 167), including the selection coefficients of missense, nonsense, and frame-shifting mutations in different cancer types, which may be useful indicators of cancer driver genes [64].

The constraint datasets that were downloaded from The Genome Aggregation Database [65] were also used to calculate the population genetic features (ID: 168 ~ 173) like LoF intolerance. The missense constraint scores are at least partially orthogonal to PolyPhen-2 scores [66]. Further information regarding these six features can be found in our previous work [22]. MutPred2 was used to evaluate the pathogenicity of all of the somatic missense mutations [67]. The combined MutPred2 score and specific functional properties were used as candidate features (ID: 174 ~ 241).

#### Genomic features.

The copy number alterations data were also download from COSMIC [56]. Copy number amplification (CNA) percentage feature (ID: 242) was calculated by the percentage of “gain” in the column of “Mut type”, whereas copy number deletion percentage (ID: 243) was calculated by 1 − CNA percentage. The enrichment of specific InterPro domains (e.g., IPR013083, Zinc finger, RING/FYVE/PHD-type) was characterized by the E-value returned by the InterProScan 5 server (https://www.ebi.ac.uk/interpro/search/sequence/). The output of InterProScan was processed to extract the E-values, followed by minus log10 transformation (ID: 244 ~ 570). The inclusion of these features in machine-learning models could test whether cancer-associated ERs were related to epigenetic and other domains. The evolution-based features, including number of human paralogs (familyMemberCount), non-coding version of the RVIS score, average non-coding GERP, primate dn/ds ratios, gene age, residual variation intolerance score, and gene damage index were also used without further processing, the description of which (ID: 571 ~ 577) can be found from the original paper [68]. The mean exon conservation phyloP (phylogenetic *P*-values) score (ID: 578) for genes with longest transcripts was also calculated by BEDOPS [69] with -mean parameter. The phyloP scores were downloaded from http://hgdownload.cse.ucsc.edu/goldenPath/hg19/phyloP46way/vertebrate/.

#### Transcriptomic features.

The gene expression data from the COSMIC database was also downloaded from the COSMIC website. The expression of COSMIC samples was averaged for each gene across different samples in same tissues to obtain median *Z* score (ID: 579 ~ 599).

#### Epigenetic features.

The data for calculating DNA methylation were derived from the processed methylation data from the COSMIC and DepMap website. For DepMap methylation data, median gene-centric methylation was calculated (ID: 601 ~ 602). For COSMIC methylation data, gene-centric differential methylation was calculated by methylation in cancer divided by methylation in normal (ID: 603 ~ 604). We downloaded the histone modification and DNase peak files (hg19) from Encyclopedia of DNA Elements (ENCODE) project [70] followed by merging adjacent peaks within 3 kb, according to the previous procedures [26]. Peak height in these data was defined by the maximum height of the merged peak. DNase and histone modification peak length and height were also calculated (ID: 605 ~ 654). S50 score for determining the median replication timing was calculated based on the algorithm from a previous study [71]. The Repli-seq Binary Alignment Map (BAM) peaks in different cell lines were downloaded from the ENCODE project website. FeatureCounts program [72] was used to assign BAM reads to gene-body of genome-wide genes. The read counts were normalized according to the sequencing depth of BAM files to obtain the S50 score (ID: 655 ~ 669). Super enhancer percentage (ID: 670 ~ 671) was calculated as the percentage of cell lines where a specific gene overlap with any super enhancers.

### Feature processing

All of the features with hg19 human genome version were downloaded if more than one genome version was available. If no hg19 version was available, features were subject to genome version conversion to hg19 by using LiftOver program. The nearest genes for specific features were found by BEDTools [73] subcommand *closest*. For numeric features, missing values were imputed with median values. For count-based features, such as gene expression counts or other similar measurements, we used zero imputation for missing values. The average values for each feature were normalized (across patients) using z-score normalization, allowing for comparison of features with different scales. Normalized features were used as the input to machine-learning models. The processed features were available in **S3 Table**.

### Training and testing cERs

Training genes are important to the cER prediction. As a matter of fact, we initially tried to build machine-learning models to differentiate cancer driver ERs from NGs. As a result, the trained models tended to predict cancer driver genes rather than cER genes. Instead, we aimed to differentiate cERs (positive labels) from non-cancer ERs (negative labels) based on various machine-learning models. Therefore, we defined the cER training and independent testing gene sets as the ERGs that were also cancer genes in at least two of the four cancer gene resources including CancerMine [29], Cancer Gene Census (CGC) database v91 [28], NCG [30], and OncoKB [32], while requiring ERGs harbouring at least a common epigenetic domain in InterPro database [74]. The epigenetic domains that we used were defined in Boukas *et al.* [5]. The ERGs with LoF mutation number (normalized by CDS length) lower than the top 20% quantile of all protein-coding genes within the common epigenetic domains (e.g., SET, JmjC, and PHD-type domains) were excluded, resulting in a total of 180 cER genes. Each of these potential genes was meticulously examined to confirm the role in cancer through epigenetic regulation. The non-cancer ERs were defined as those ERGs that were also in the NG list, resulting in a total of 94 ERs as negative genes. The training and testing genes were chosen from these genes, adhering to an 8:2 ratio (**Figs 1** and **S1**). The division between training and test sets has minimal impact on the model. The cER genes were further classified into distinct subtypes—reader, writer, eraser, and remodeler—utilizing information from the CR2Cancer database [54] for subtype-specific prioritization. The final gene definitions are detailed in **S2 Table**.

### Machine-learning model evaluation

The primary aims of this study are to separately generate ranking scores to prioritize cER genes and the four types of cERs, based on a better machine-learning model. We tried different machine-learning models to do this task, including three supervised models (SVM, RF, and XGBoost), four semi-supervised machine-learning models (TSVM, co-training, tri-training, and SemiBoost), and four semi-supervised deep-learning models (FlexMatch, MixMatch, LadderNetwork, and UDA). Semi-supervised models, consisting of supervised and unsupervised learning approaches, leverages data from both labeled and unlabeled genes (genes but not training, or testing genes) to enhance model training. A semi-supervised dataset typically comprises a small proportion of data with labeled genes alongside a larger number of unlabeled genes. This kind of models is particularly suitable to the circumstance that only a limited number of labeled genes is available for model training, which can be less resource-intensive and less complex than fully supervised learning and may have better performance than unsupervised learning. The chosen 11 models were trained through extensive hyperparameter tuning to ensure their robustness in independent testing and final prediction. The hyperparameters used for different models were shown in **S9 Table**. The deep-learning models employed a one-dimensional ResNet50 as their backbone network. Further details on the specific parameter settings for different models can be found in **S10 Table**. For benchmarking, we also used the GCN module released with EMOGI [75] (https://github.com/schulter/EMOGI) and the reference implementation of GAT (https://github.com/PetarV-/GAT). Because both models operate in an end-to-end manner, multi-omic gene characteristics were concatenated to form the initial node feature vectors. GraphSAGE (https://arxiv.org/abs/1706.02216), which samples and aggregates neighbourhood information through trainable pooling functions rather than attention or fixed convolutional weights, was also used.

Ten-fold CV was used to primarily evaluate the performance of the models (Table 1). The machine-learning metrics used in model performance evaluation includes accuracy, area under the AUROC, AUPRC, F1 score, precision, and recall. To reduce the potential error resulting from the varying sample sizes, each model employed a SMOTE (Synthetic Minority Over-sampling Technique) process. The mean and standard deviation were calculated for the results from the ten folds of CVs. The independent testing for these models was evaluated based on the randomly chosen testing gene set. Our various trials indicated that the different gene set partitioning exerted negligible influence on predictive performance. The areas under the receiver operating characteristic curve (ROC) and the precision-recall curve (PRC) were used to select the major model used in the CASER approach.

### The tri-training model

Supervised learning models typically require a large amount of labeled data for effective training. However, the number of known cancer-driving ERGs is limited. Semi-supervised models offer a theoretically robust solution to this challenge. The tri-training model constructs three classifiers using an initial set of labeled genes (training cER genes) and iteratively improves their performance by incorporating unlabeled instances (other ERGs) during training [76].

Tri-training is an ensemble-based semi-supervised learning approach that utilizes three base models to enhance generalization. It iteratively selects the most confident predictions from each model to label additional data, thereby reducing the risk of overfitting and improving overall model robustness. The core principle of the tri-training model is summarized as follows: Initially, three base SVM classifiers (parameters were shown in **S10 Table**) are constructed by randomly sampling training data from a single-view labeled dataset (*L*) using the bootstrap method (**S13 Fig**). SVM classifiers were chosen due to their superior performance in the evaluation. The training set for each base classifier is then iteratively expanded. If two of the three base classifiers generate identical predictions for an unlabeled gene (*x*) from the unlabeled dataset (*U*), this gene, along with its pseudo-label, is added to the training set of the third classifier. This process increases the likelihood that the pseudo-labeled data will positively influence the training of the classifiers. The model’s robustness stems from using the agreement of two models to determine whether to incorporate unlabeled genes, rather than relying solely on the confidence score of a single model. The iterative process continues, with classifiers being updated at each step, until the error rate of the base classifiers stabilizes, marking the completion of training. Unlike other self-training methods, where misclassified unlabeled genes may persist in the training set and negatively affect learning, the tri-training model re-selects the unlabeled dataset and its pseudo-labels in each iteration. Notably, if both base classifiers exhibit high prediction error rates, they forgo the use of unlabeled data, even if their predictions are consistent. This built-in mechanism enhances the reliability of the semi-supervised model and effectively mitigates performance degradation caused by incorrect pseudo-labelling.

cER prioritization by the proposed CASER approach

We employed the tri-training model to develop a predictive approach, termed CASER. An overview of the CASER approach is illustrated in **S3 Fig**. To address class imbalance, we utilized the borderline SMOTE technique [77]. To ensure robust predictions, we assigned a *p*-value to each gene using a previously described Monte Carlo simulation method [55]. For the feature profile, the Monte Carlo simulation was performed 100 times, with all features recomputed in each iteration. Next, each “simulated” gene was scored with the previously trained CASER model on the genuine data, generating a set of cER scores that formed an empirical null distribution. The *p*-value for each gene was calculated as the fraction of scores from the simulations equal to or exceeding the observed score. To correct for multiple hypothesis testing, we applied the Benjamini-Hochberg method to obtain *q*-values. A gene was considered statistically significant if its *q*-value was < 0.1. The independent prediction of four types of cERs was performed independently in the same manner. The cancer-specific prioritization of cERs was conducted using cancer-specific feature profiles, following the same procedure as the pan-cancer prioritization.

### Gene sets for evaluation

We used several cancer driver gene annotations from different databases to evaluate the CASER prediction, including (1) CancerMine, a web-based tool that extracted text mining from literature and reported cancer drive genes across cancers [29]; (2) CGC v91, an expert-curated cancer driver gene list [28]; (3) NCG, a manually curated cancer gene set based on CGC and literature [30]; (4) IntOGen (v2023.05.31), a database including the cancer driver genes predicted from the mutational data derived from sequenced tumor samples of patients [31]; and (5) OncoKB, a precision oncology knowledge that includes curated cancer genes and related clinical resource [32]. FACER [24], a tool that can also identify cancer-associated ERs based on TCGA multi-omics data, identified 225 cERs. However, these cERs were not strictly validated in the original study. These genes were used as a comparison to the CASER-predicted cERs in prediction evaluation.

### Functional genomic datasets for evaluating the predicted cERs

We used the CRISPR or shRNA screen data to further evaluate the cancer driving potential of the prioritized cERs. These datasets are instrumental in prioritizing and evaluating essential genes and cancer driver genes by analysing tumor cell line viability post-gene silencing and have been extensively employed for the assessment of cancer driver genes [22,78]. The data that we used includes: (1) The EPIKOL data, an ER gene-based CRISPR screen dataset, which was used to identify ER gene vulnerabilities [20]; The robust rank aggregation (RRA) scores of negatively selected genes, as calculated by MAGeCK in the original study [45], were used in evaluation. A higher RRA score indicates a more pronounced effect on cell survival following the knockdown of the corresponding gene. (2) The shRNA screen (File name: Achilles_v2.4.6.rnai.gct) data, which were downloaded from the DepMap website (https://depmap.org/portal/data_page/?tab=overview); (3) The CRISPR screen version 24Q4 (File name: CRISPRGeneDependency.csv) data from the DepMap website. The shRNA screen data was analyzed by DEMETER2 algorithm [79], while the CRISPR screen dependency value was estimated by Chronos algorithm [44], in different cell line. The DepMap data offered novel insights derived from a large-scale loss-of-functional screening for tens of thousands of human protein-coding genes across a large number of cancer cell lines, utilizing either shRNA knockdown or CRISPR knockout technologies [43]. Specifically, the CRISPR screening dataset encompasses 1,095 cell lines, while the shRNA screening dataset includes 216 cell lines. However, the samples in the earlier version of the CRISPR screen data used in training were not used in the evaluation of the prediction results. The gene-centric dependency score was averaged by median values for different cell lines in the three datasets; (4) The precomputed survival data from the OncoRank website (http://www.oncolnc.org/) was used to evaluate the survival of cancer patients for different gene sets.

### Network module analysis

For the CASER-predicted cER genes, the module analysis was done based on the pharmacogenomic database, PharmacoDB [48]. The MCODE algorithm [49] was applied to identify densely connected network components. The parameters of MCODE included degree cutoff of 2, node score cutoff of 0.2 and max depth of 100, haircut option is true. The network was visualized by Cytoscape software (version 3.10.1) [80].

### Gene-set enrichment analysis

The “aPEAR” R package [39] was used to perform and summarize the results of GSEA, leveraging the similarities between the significantly enriched GOBP pathways with a minimum cluster of size of 5. The enriched pathways were visualized by the “enrichmentNetwork” function in “aPEAR” package. Each cluster was assigned a biologically meaningful label by aPEAR. Gene set enrichment analyses for the KEGG and DisGeNET [41] gene set were done using Enrichr [81].

### Cell culture and transfection

The human melanoma cell line SK-mel-2, clear cell renal cell carcinoma cell line Caki-1, breast cancer cell line MDA-MB-231, and prostate cancer cell line LNCaP, along with their corresponding complete median, were purchased from Procell Life Science & Technology Co., Ltd. (Wuhan, China). All cell lines were grown in complete medium and incubated at 37 °C in a humidified atmosphere of 5% CO_2_. The cells were seeded at a density of 1 × 10^6^ cells/well in 6-well plate, and then grown for 24 hr prior to transfection. Single-stranded small interfering RNA (siRNA) targeting the investigated human genes and the non-targeting control (NC) siRNA duplexes were chemically synthesized by Biosystems (General Biosystems, Anhui, China) using standard phosphoramidite chemistry. 7.5 µL siRNAs (20 µM) were prepared and mixed with 7.5 µL Lipofectamine 3000 transfection reagent (#L3000075, Invitrogen, USA). The mix was transfected into both of the two cell lines using Opti-MEM I reduced serum medium (#31985070, Invitrogen, USA). This medium was replaced by growth medium at 6 ~ 8 hr after transfection. At 48 hr after transfection, the cells were collected and used for the following experiments.

### Quantitative reverse transcription PCR (qRT-PCR)

Total RNA was isolated using Trizol (Tiangen) protocol. The concentration of RNA was determined using the NanoDrop (Nano-200 Micro-Spectrophotometer, Hangzhou, China). Then, 1 μg of total RNA was reverse transcribed to complementary DNA (cDNA) using RevertAid Reverse Transcriptase (#EP0441, Thermo), according to the manufacturer’s protocol. qRT-PCR was performed on ABI Q1 qPCR instrument using the PerfectStart Green qPCR SuperMix (#AQ601–04, Takara). The sequences of the primers used were listed in **S11 Table**. The mRNA expression of each investigated gene was normalized to that of *GAPDH*. Gene expression levels were quantified as threshold cycle (Ct) values, while relative quantification levels of genes were normalized with *GAPDH* using the 2^−ΔΔ𝐶𝑇^ cycle threshold method.

### CCK-8 assay

The proliferation in cell lines was measured using CCK-8 kit (#C0037, Beyotime, China). MDA-MB-231 and SK-mel-2 cells were seeded at 3,000 cells per well in 96-well plates, while Caki-1 and LNCaP cells were seeded at 2,000 cells per well in 96-well plates with 100 μL complete medium. The cell lines were incubated at 37 °C (5% CO_2_) for 24 hr to the logarithmic growth period, 24, 48, and 72 hrs after culture, and then 10 μL CCK-8 reagent was added, and then cells were cultured in the original medium for 1.5 hr, respectively. After that, the absorbance at 450 nm was calculated for cell viability. Finally, the colorimetric measurements of optical density (OD) value were detected by a microplate reader at 450 nm (#ELx800, BioTek, United States).

### Statistics

Statistical analysis of bar or line plots for molecular experiments was carried out with using R software. Results are represented as the mean ± standard error of the mean (SEM). One-way analysis of variance (ANOVA) followed by Dunnett’s corrections was used to analyze the difference between two groups.

Statistical significance in box plots was determined by one-tailed or two-tailed Wilcoxon rank-sum test. *P*-values were indicated by star symbol, *, *P* < 0.05; **, *P* < 0.01; ***, *P* < 0.001; ****, *P* < 0.0001. Not significant *p*-value was indicated by n.s. (not significant). In machine-learning prediction, *p*-value was adjusted by the false discovery rate method.

### The testing environment for machine learning models

In this paper, the machine-learning models were tested on a Linux server with Intel(R) Xeon(R) Gold 5220R CPU @ 2.20GHz and a single GeForce RTX 3090 (NVIDIA) graphics processing unit of 24 GB. The major software used in machine-learning included Python v3.9.16, Pytorch v1.11.0, imbalanced-learn v0.12.3, scikit-learn v1.2.1, and a semi-supervised learning package LAMDA-SSL v1.0 [82].

## Supporting information

S1 Fig Performance of different machine-learning and deep-learning models on the testing gene set. (A) The area under the receiver operating characteristic curve (AUROC) for three machine-learning models. (B) The area under the precision-recall curve (AUPRC) for three machine-learning models. (C) The AUROC for three models based on deep graph neural network architecture. (D) The AUPRC for three models based on deep graph neural network architecture.
(TIF)

S2 Fig Performance evaluation of CASER model variants under varying downsampling ratios. (A) The area under the receiver operating characteristic curve (AUROC). (B) The area under the precision-recall curve (AUPRC).
(TIF)

S3 Fig Technical demonstration of the CASER model. (TIF)

S4 Fig Performance of the CASER model with different feature subsets on the testing gene set. (A) The area under the receiver operating characteristic curve (AUROC) for CASER with the indicated feature subset. (B) The area under the precision-recall curve (AUPRC) for CASER with the indicated feature subset. (C) The AUROC for CASER without specific feature subset. (D) The AUPRC for CASER without specific feature subset. The dashed line represents theoretical values.
(TIF)

S5 Fig Bar plot illustrates the absolute mean SHapley Additive exPlanations (SHAP) values. Features are ordered by absolute SHAP values. Top 32 features are shown due to limited space.
(TIF)

S6 Fig The Circos plot displays the predicted pan-cancer cER genes and subtype-specific cER genes. The outermost ring contains the corresponding gene symbols.
(TIF)

S7 Fig Boxplots showing the ATAC-seq peak score measuring open chromatin for for non-cancer ERs, unpredicted ERs, CASER-predicted novel cERs, and known cERs. (A) ATAC-seq peak score from pan-cancer data. (B)-(E) ATAC-seq peak score from four representative TCGA cancer types. *P*-values are shown in the plots and are calculated by Wilcoxon rank-sum two-tailed test.
(TIF)

S8 Fig Boxplots showing the Cox hazard ratio (HR) score for different gene groups in nine representative TCGA cancer types. BRCA, Breast invasive carcinoma; GBM, Glioblastoma multiforme; ESCA, Esophageal carcinoma; CESC, Cervical squamous cell carcinoma and endocervical adenocarcinoma; KIRP, Kidney renal papillary cell carcinoma; LAML, Acute Myeloid Leukemia; PAAD, Pancreatic adenocarcinoma; SARC, Sarcoma; OV, Ovarian serous cystadenocarcinoma. Unpredicted, ERs that are not predicted as cERs; NGs, neutral genes. *P*-values are shown in the plots and are calculated by Wilcoxon rank-sum two-tailed test.
(TIF)

S9 Fig The relative mRNA expression of six candidate genes in two cancer cell lines, as determined by qRT-PCR. NC, the non-targeting siRNA, was used as the negative control.
(TIF)

S10 Fig Investigation of six candidate cER genes in affecting the proliferation of two cancer cell lines. (A) Six predicted cER genes were investigated in MDA-MB-231 cell line (*n* = 4). (B) Six predicted cER genes were investigated in LNCaP cell line (*n* = 4).
(TIF)

S11 Fig Dual-role cER genes are overrepresented in the PharmacoDB gene-medicine networks. (A) Complete single/dual-role cERs and medicine bipartite network. (B) The Molecular Complex Detection (MCODE) algorithm is applied to the bipartite network to identify densely connected network modules (or backbones). Gene categories are colors coded based on the legend.
(TIF)

S12 Fig Distribution of gene degrees for different gene sets in the gene-medicine network from the PharmacoDB database. Dual-role and single-role cERs are from (A) novel cERs, (B) known cERs, and (C) all-predicted cERs. Nonpredicted ERs and neutral genes are shown in (D). Dashed line represents mean node degree. *P*-value is calculated by Wilcoxon rank-sum one-tailed test.
(TIF)

S13 Fig Demonstration of tri-training model. This figure was generated by Figdraw (www.figdraw.com) tool.
(TIF)

S1 Table Summary of the features used in machine-learning models. (XLSX)

S2 Table ERG (Epigenetic regulator gene) annotation. (XLSX)

S3 Table Processed features used in machine-learning models. (XLSX)

S4 Table Predictive performance of tri-training models utilizing various SVM kernel configurations on the independent testing set. (XLSX)

S5 Table Prediction results of the Cancer-ASsociated Epigenetic Regulator identification (CASER) approach. (XLSX)

S6 Table List of the novel cER (cancer-associated epigenetic regulator) genes as well as the subtypes. Genes shown here are excluded from that from known cER genes.
(XLSX)

S7 Table List of the 80 novel cER genes that are not available as cancer genes in the four cancer gene databases. (XLSX)

S8 Table Cancer-specific prediction results of the Cancer-ASsociated Epigenetic Regulator identification (CASER) approach. (XLSX)

S9 Table Summary of the hyperparameters for tuning different machine-learning models. (XLSX)

S10 Table Summary of the hyperparameters used in the different models. (XLSX)

S11 Table Summary of the qRT-PCR primers used in this study. (XLSX)
