# Supplementary figures and images for: CASER: A semi-supervised model with multi-omics data integration prioritizes cancer-associated epigenetic regulator genes
Source: PLoS Comput Biol. 2026 Apr 28;22(4):e1014253. doi: 10.1371/journal.pcbi.1014253 (PMC13138745; doi:10.1371/journal.pcbi.1014253)

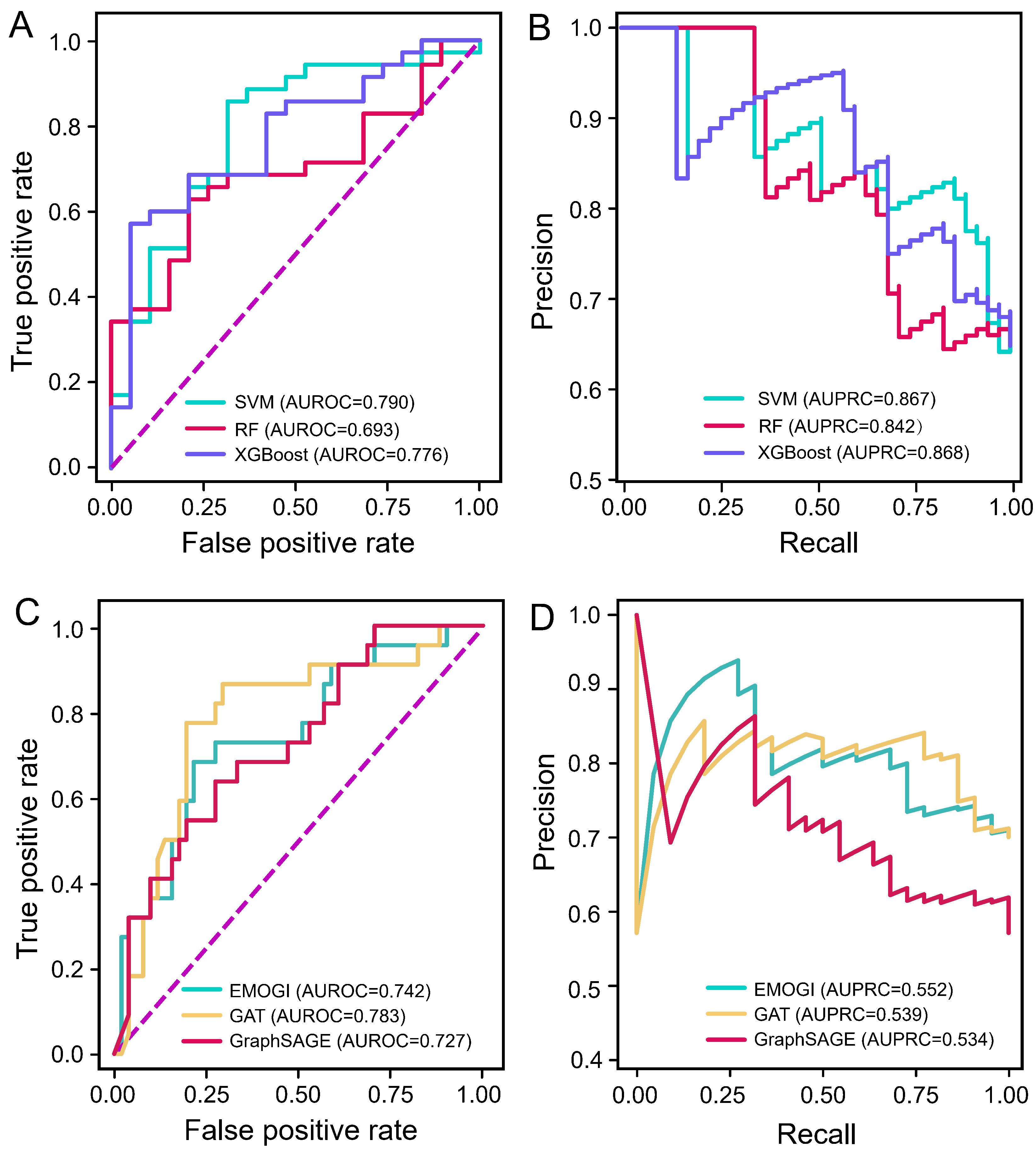

Supplement: S1 Fig — (A) The area under the receiver operating characteristic curve (AUROC) for three machine-learning models. (B) The area under the precision-recall curve (AUPRC) for three machine-learning models. (C) The AUROC for three models based on deep graph neural network architecture. (D) The AUPRC for three models based on deep graph neural network architecture. (TIF) [file pcbi.1014253.s001.tif]

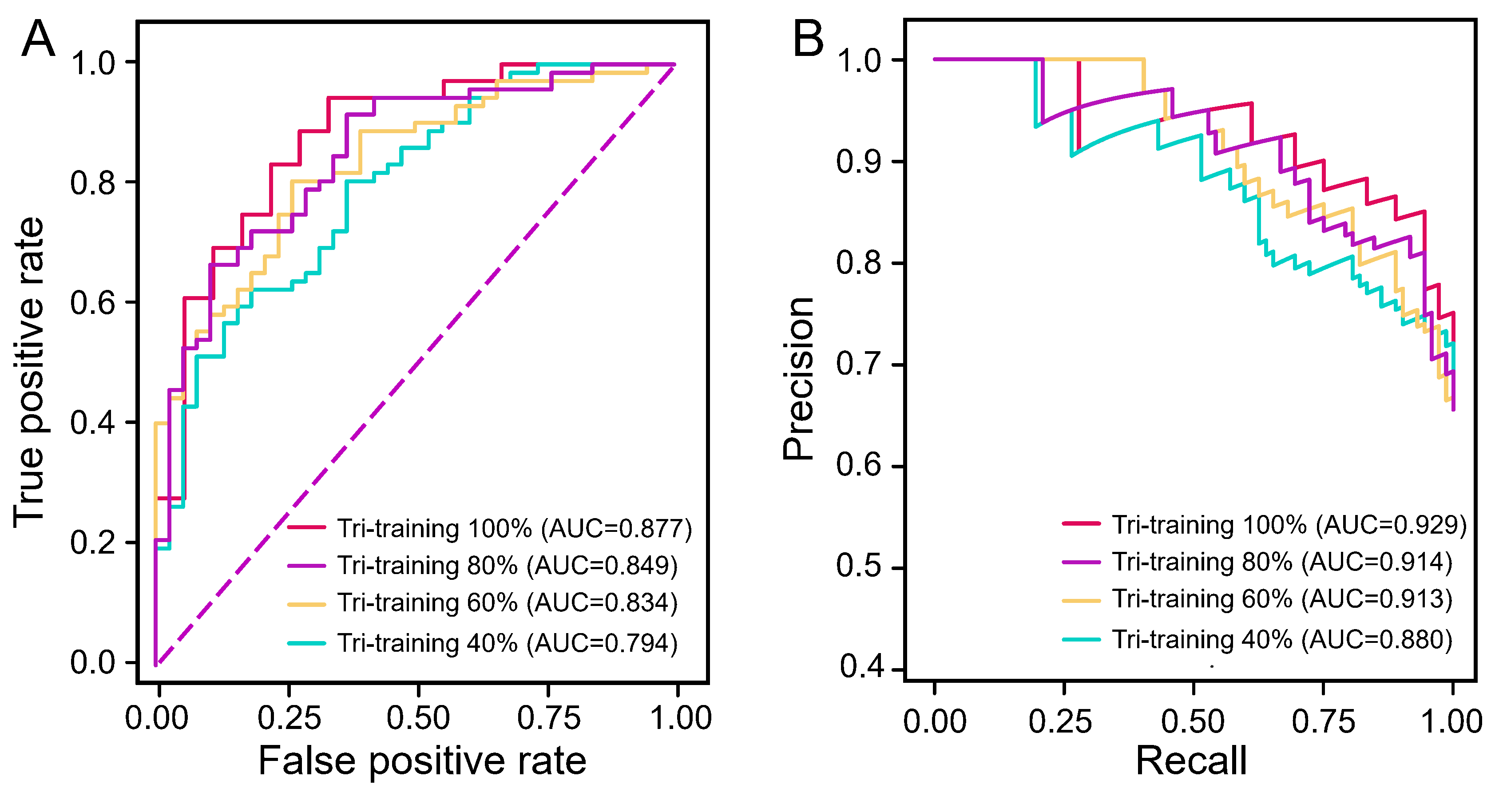

Supplement: S2 Fig — (A) The area under the receiver operating characteristic curve (AUROC). (B) The area under the precision-recall curve (AUPRC). (TIF) [file pcbi.1014253.s002.tif]

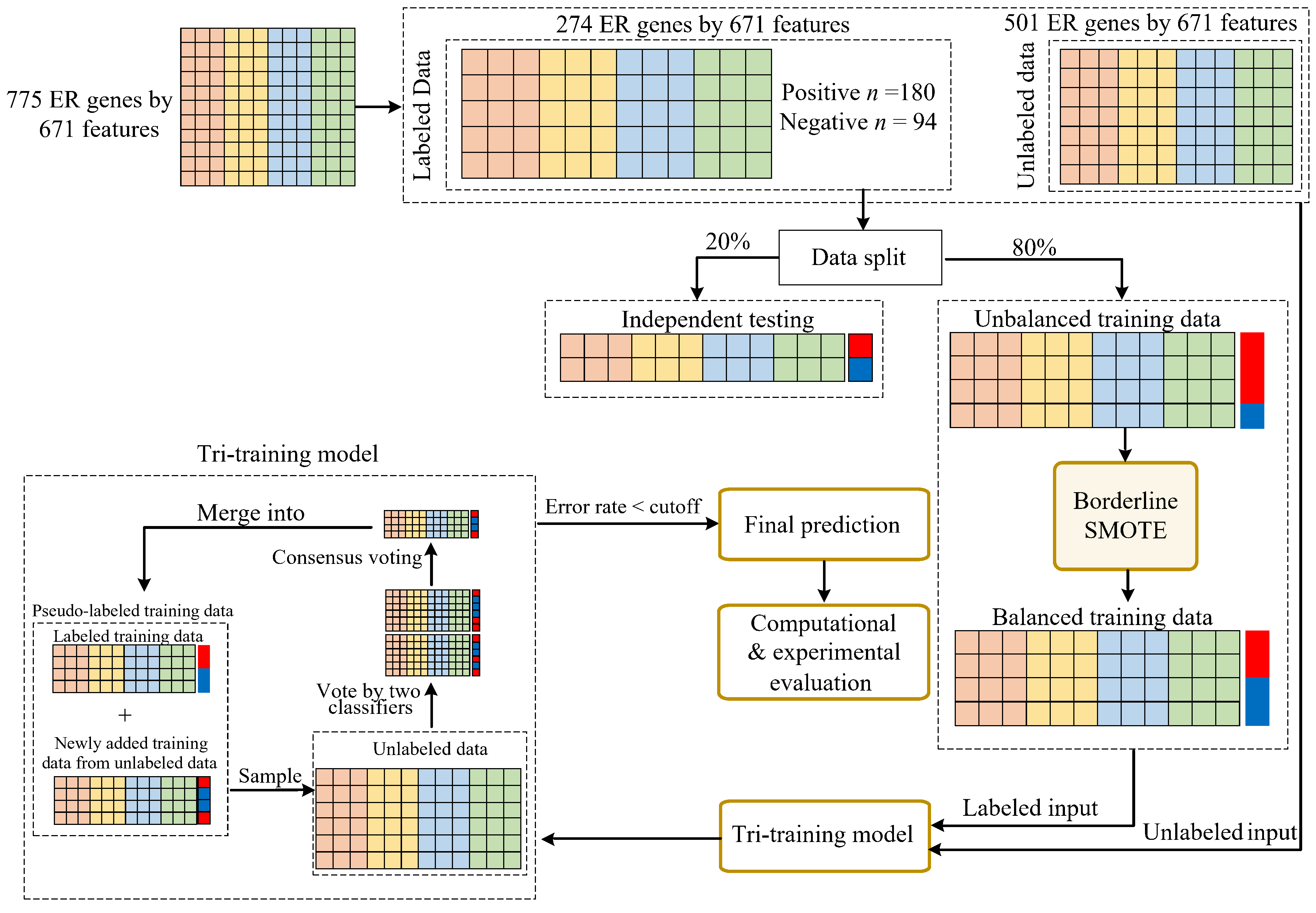

Supplement: S3 Fig — (TIF) [file pcbi.1014253.s003.tif]

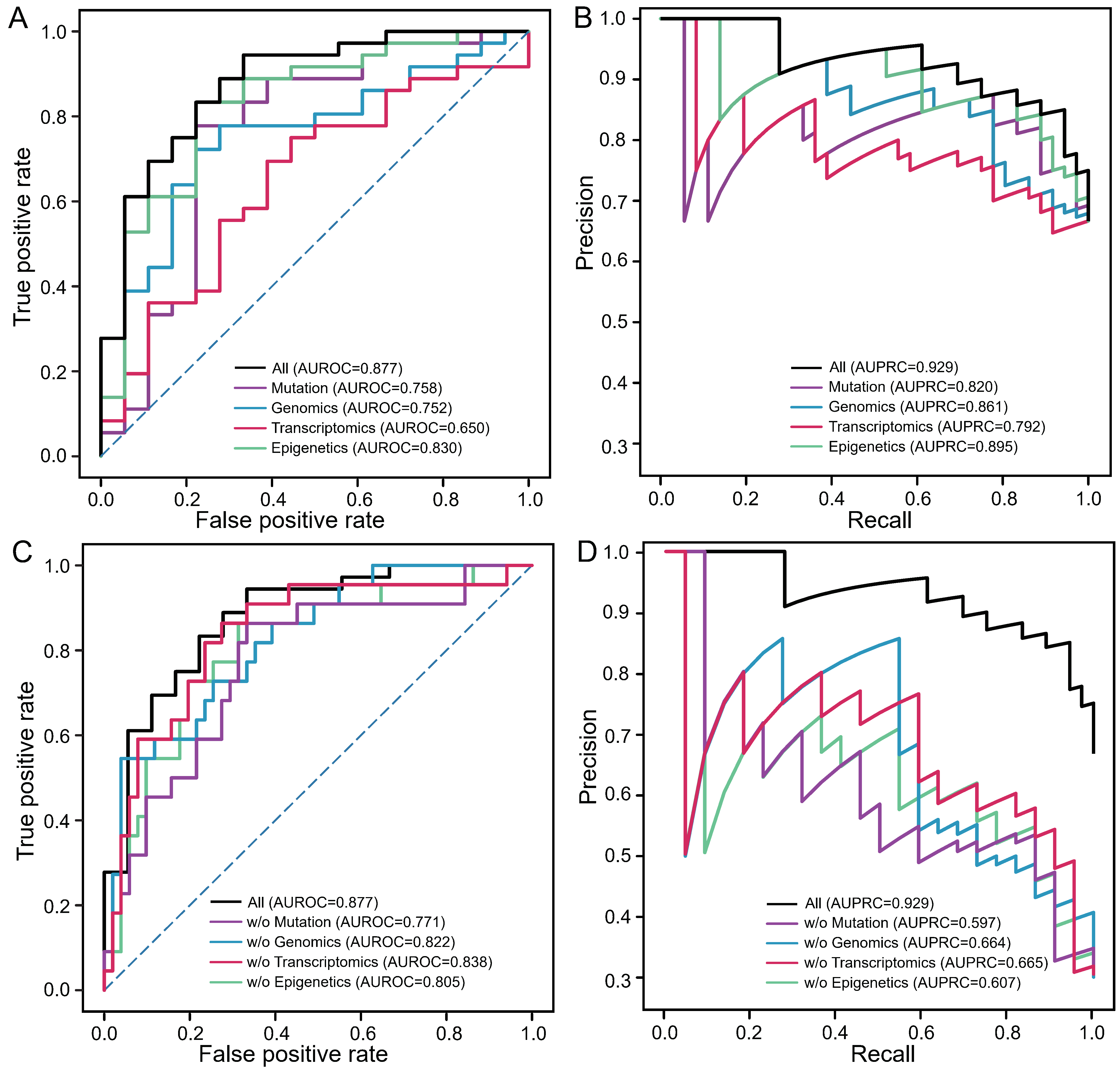

Supplement: S4 Fig — (A) The area under the receiver operating characteristic curve (AUROC) for CASER with the indicated feature subset. (B) The area under the precision-recall curve (AUPRC) for CASER with the indicated feature subset. (C) The AUROC for CASER without specific feature subset. (D) The AUPRC for CASER without specific feature subset. The dashed line represents theoretical values. (TIF) [file pcbi.1014253.s004.tif]

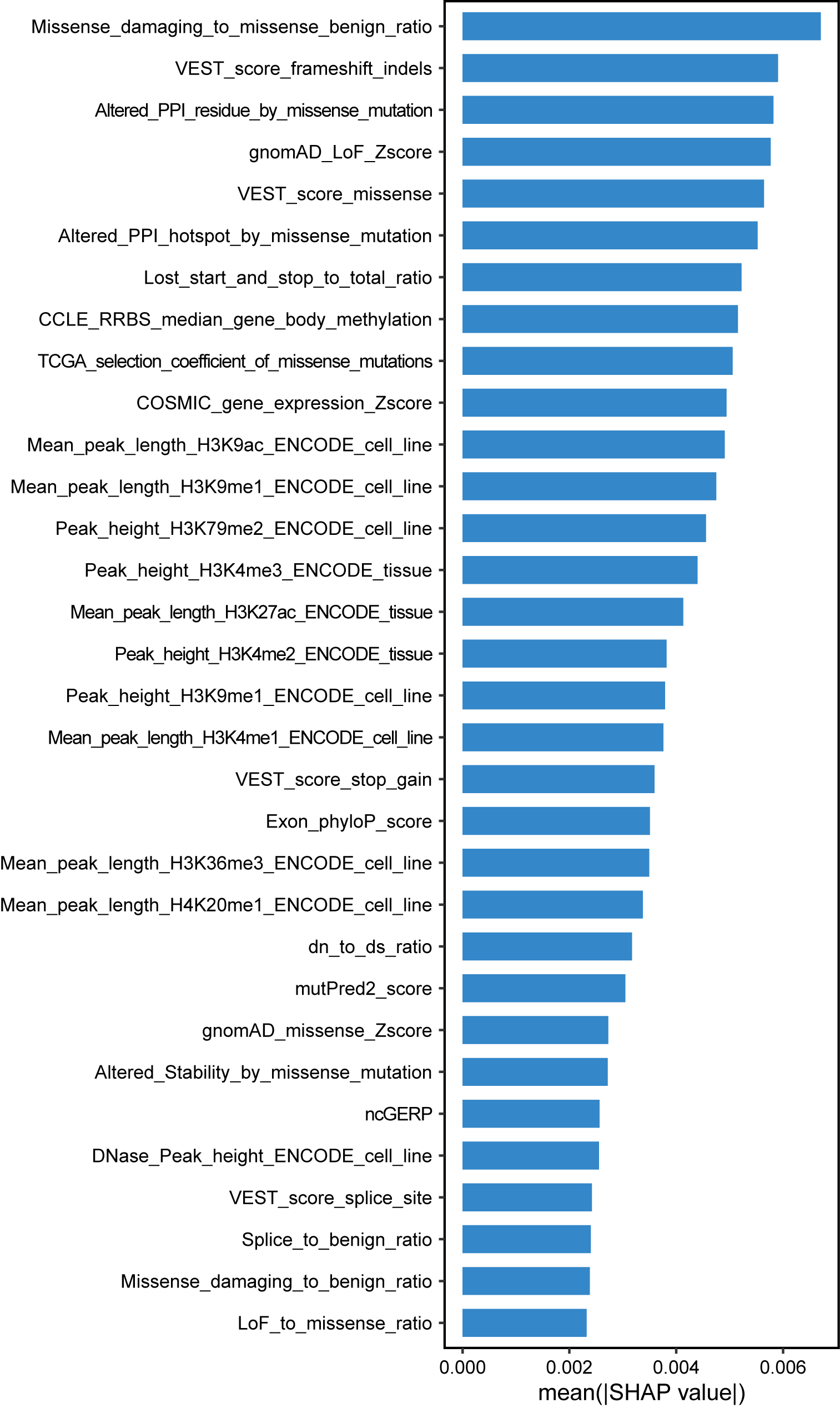

Supplement: S5 Fig — Features are ordered by absolute SHAP values. Top 32 features are shown due to limited space. (TIF) [file pcbi.1014253.s005.tif]

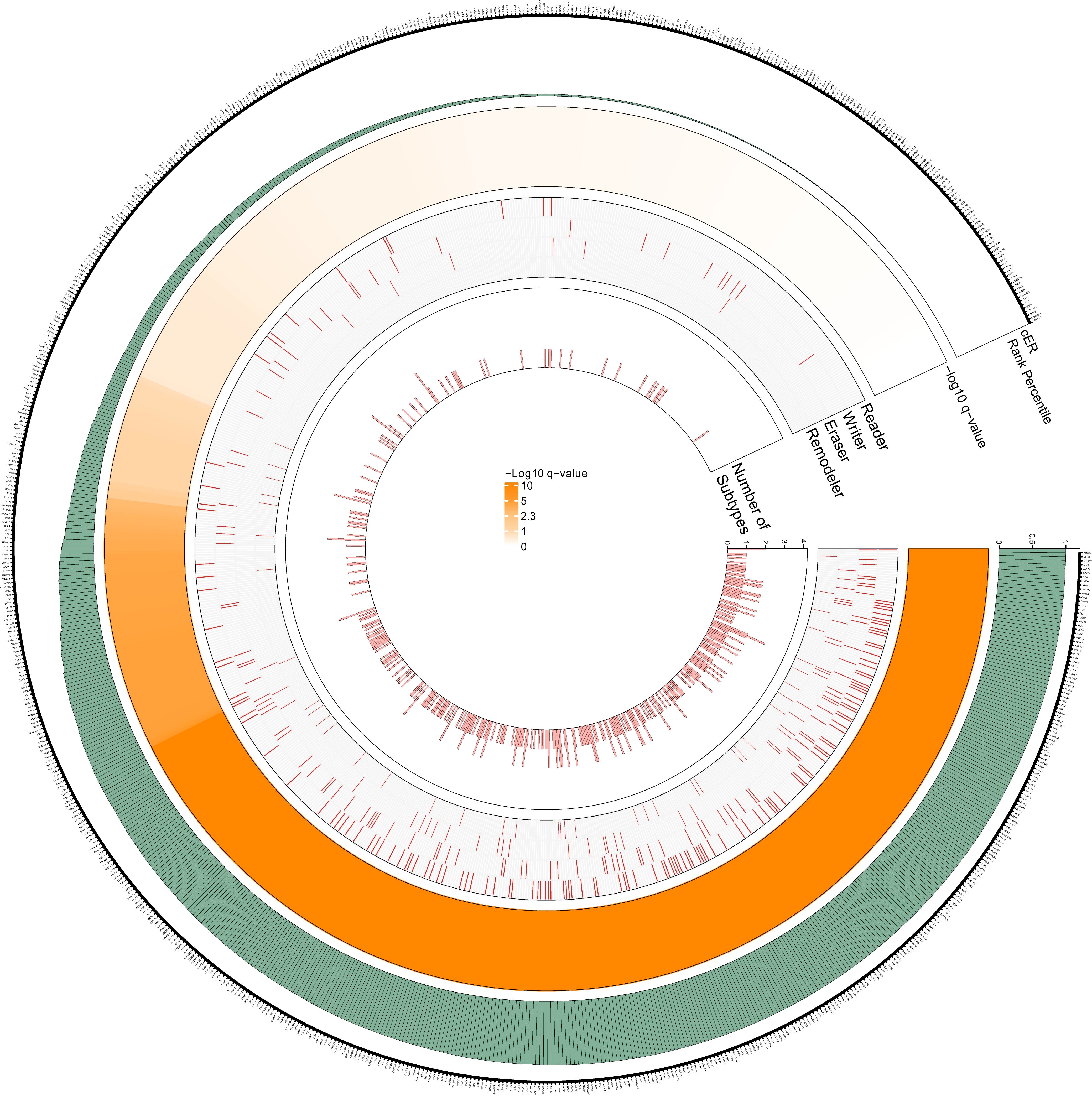

Supplement: S6 Fig — The outermost ring contains the corresponding gene symbols. (TIF) [file pcbi.1014253.s006.tif]

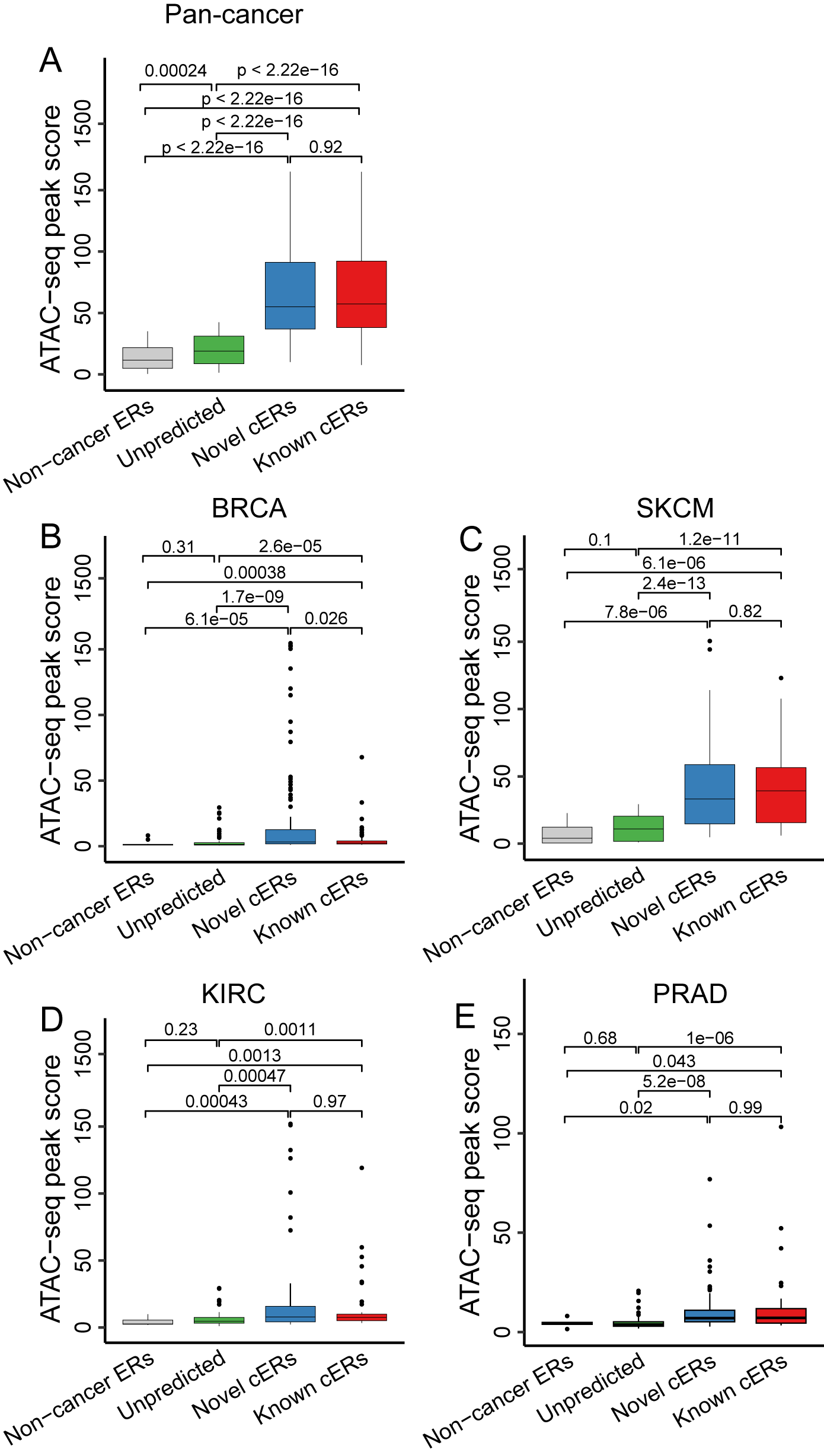

Supplement: S7 Fig — (A) ATAC-seq peak score from pan-cancer data. (B)-(E) ATAC-seq peak score from four representative TCGA cancer types. P-values are shown in the plots and are calculated by Wilcoxon rank-sum two-tailed test. (TIF) [file pcbi.1014253.s007.tif]

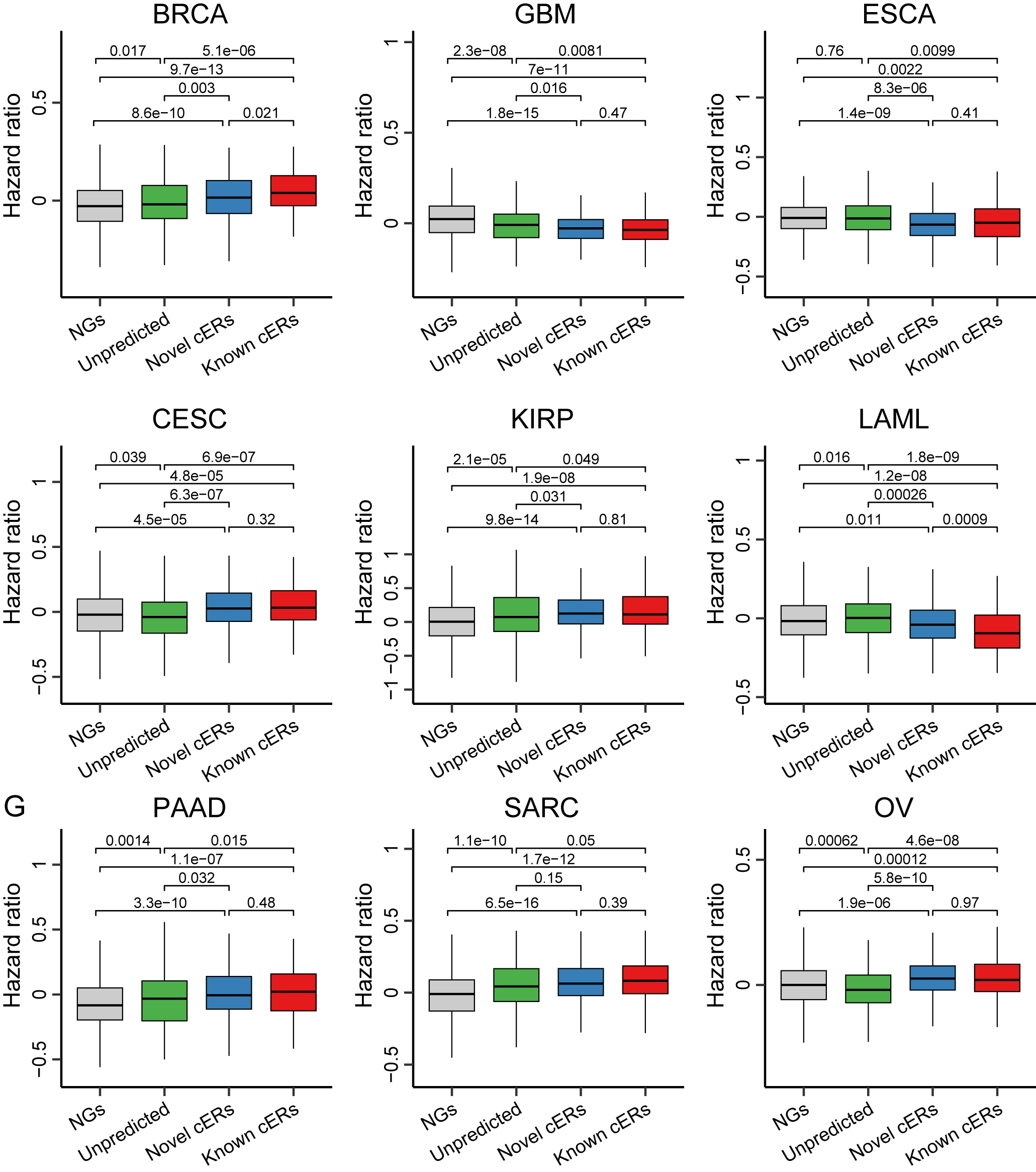

Supplement: S8 Fig — BRCA, Breast invasive carcinoma; GBM, Glioblastoma multiforme; ESCA, Esophageal carcinoma; CESC, Cervical squamous cell carcinoma and endocervical adenocarcinoma; KIRP, Kidney renal papillary cell carcinoma; LAML, Acute Myeloid Leukemia; PAAD, Pancreatic adenocarcinoma; SARC, Sarcoma; OV, Ovarian serous cystadenocarcinoma. Unpredicted, ERs that are not predicted as cERs; NGs, neutral genes. P-values are shown in the plots and are calculated by Wilcoxon rank-sum two-tailed test. (TIF) [file pcbi.1014253.s008.tif]

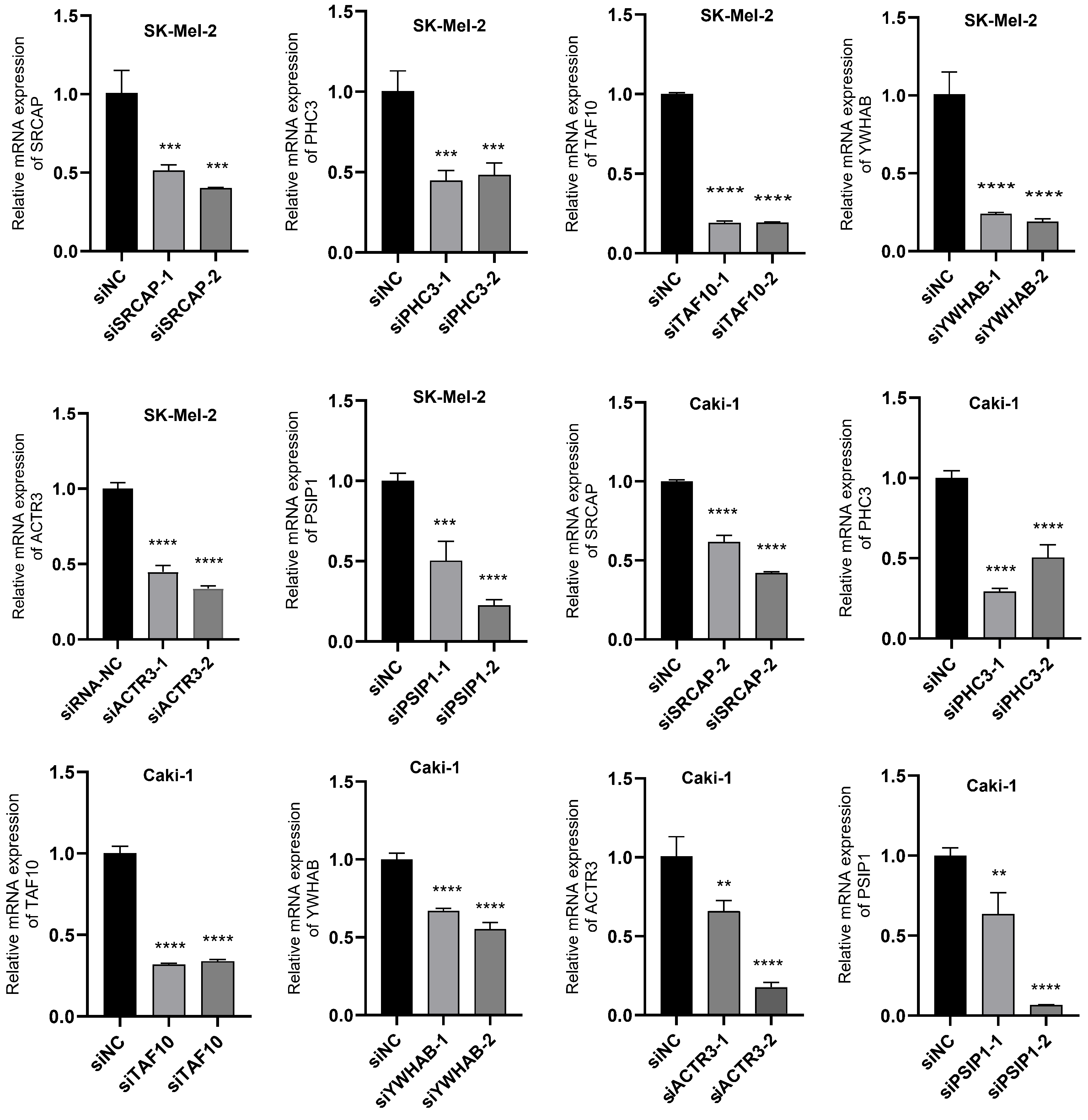

Supplement: S9 Fig — NC, the non-targeting siRNA, was used as the negative control. (TIF) [file pcbi.1014253.s009.tif]

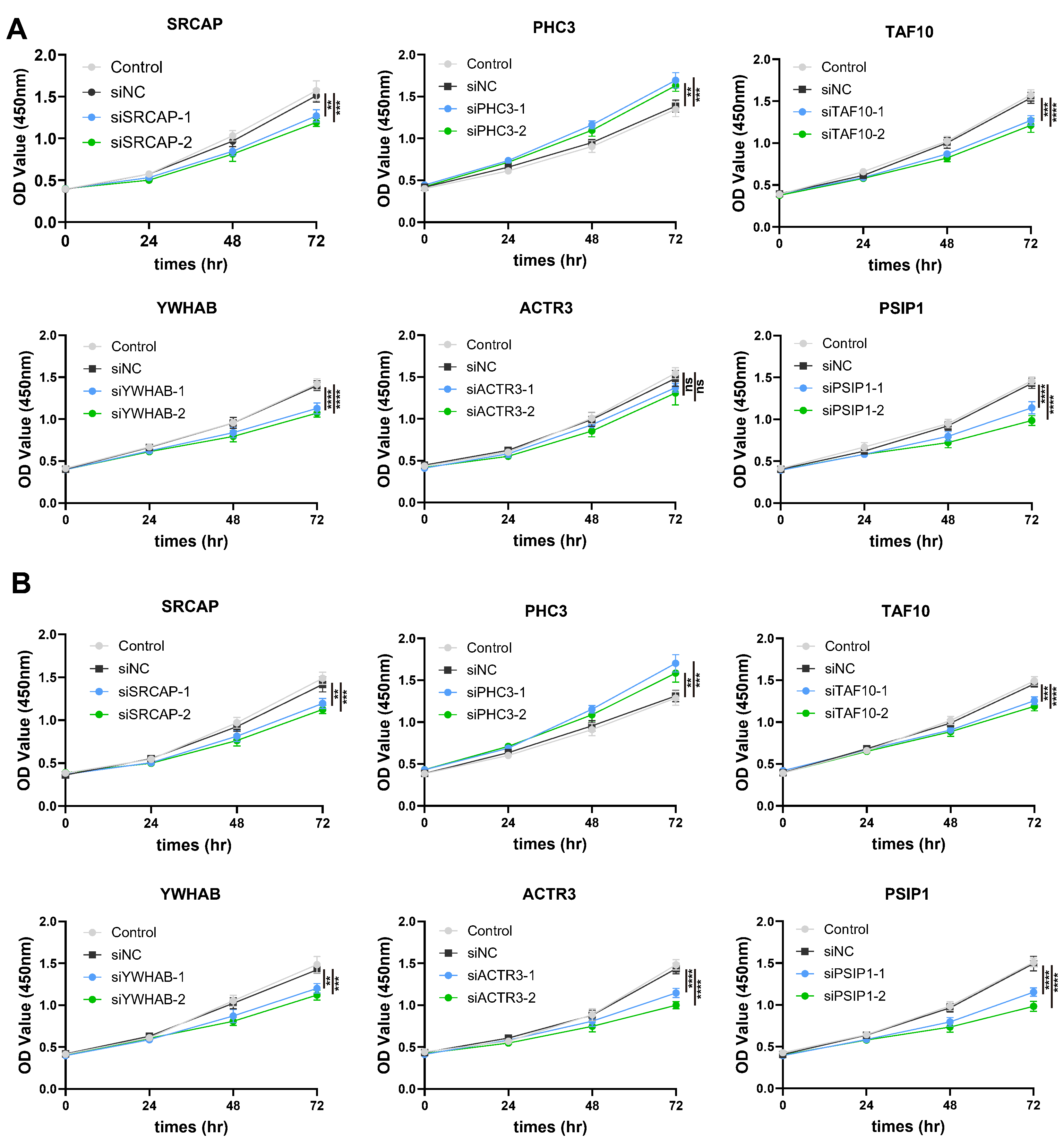

Supplement: S10 Fig — (A) Six predicted cER genes were investigated in MDA-MB-231 cell line (n = 4). (B) Six predicted cER genes were investigated in LNCaP cell line (n = 4). (TIF) [file pcbi.1014253.s010.tif]

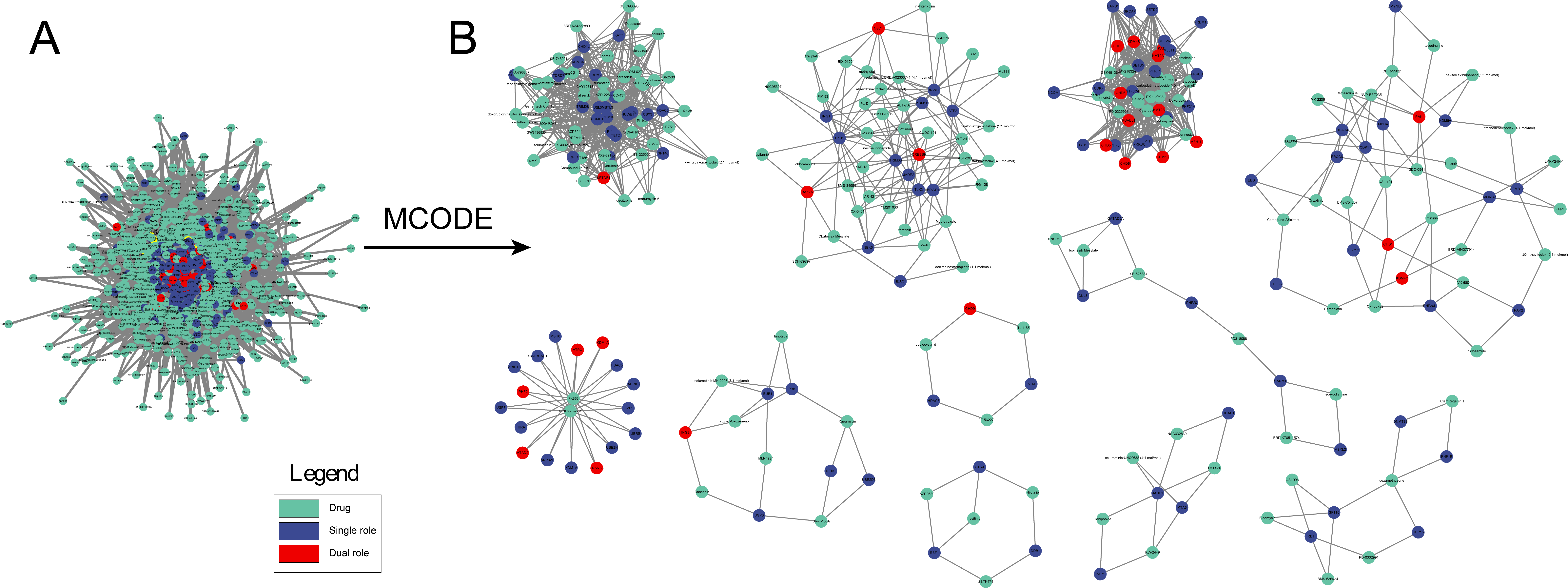

Supplement: S11 Fig — (A) Complete single/dual-role cERs and medicine bipartite network. (B) The Molecular Complex Detection (MCODE) algorithm is applied to the bipartite network to identify densely connected network modules (or backbones). Gene categories are colors coded based on the legend. (TIF) [file pcbi.1014253.s011.tif]

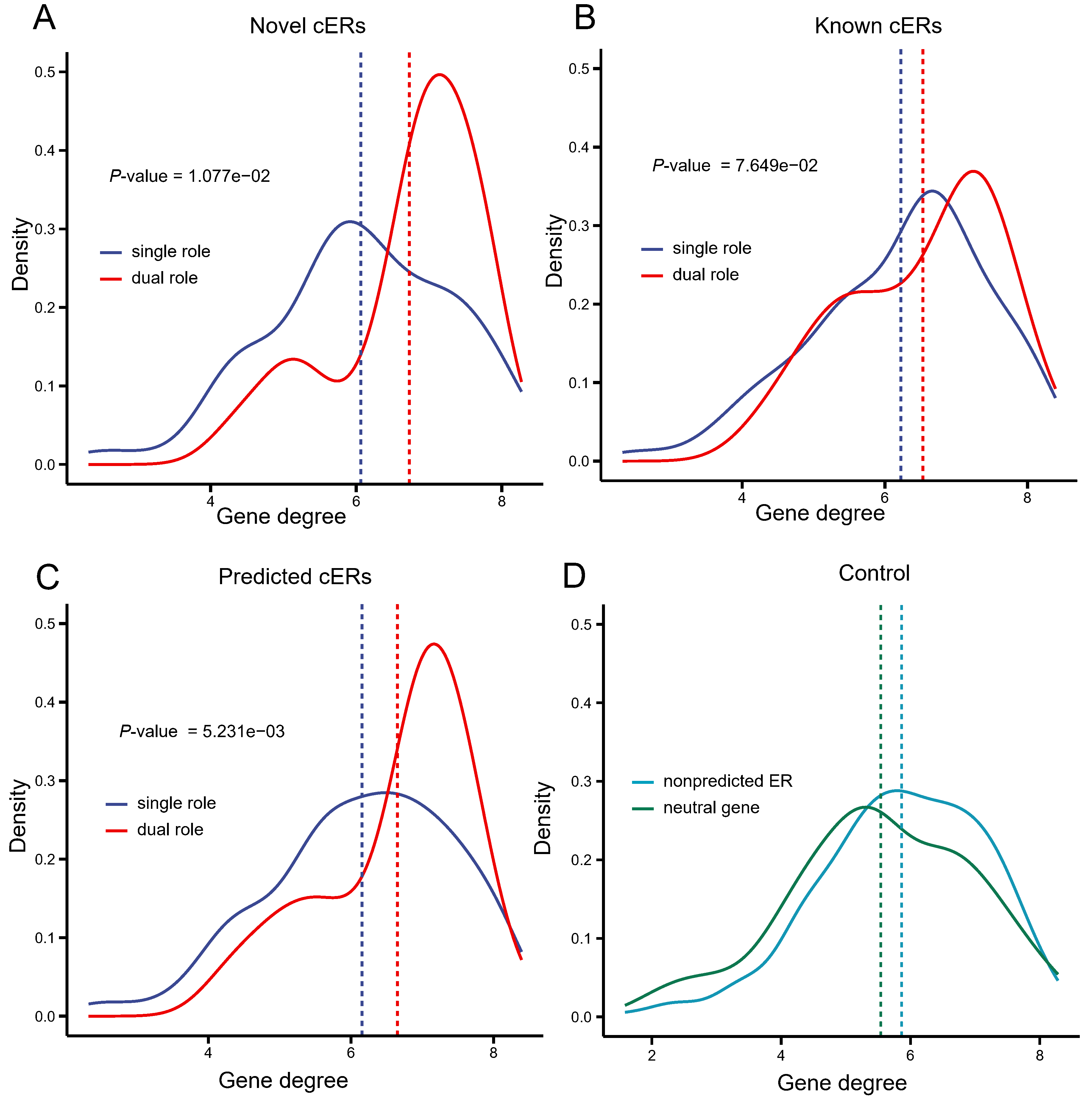

Supplement: S12 Fig — Dual-role and single-role cERs are from (A) novel cERs, (B) known cERs, and (C) all-predicted cERs. Nonpredicted ERs and neutral genes are shown in (D). Dashed line represents mean node degree. P-value is calculated by Wilcoxon rank-sum one-tailed test. (TIF) [file pcbi.1014253.s012.tif]

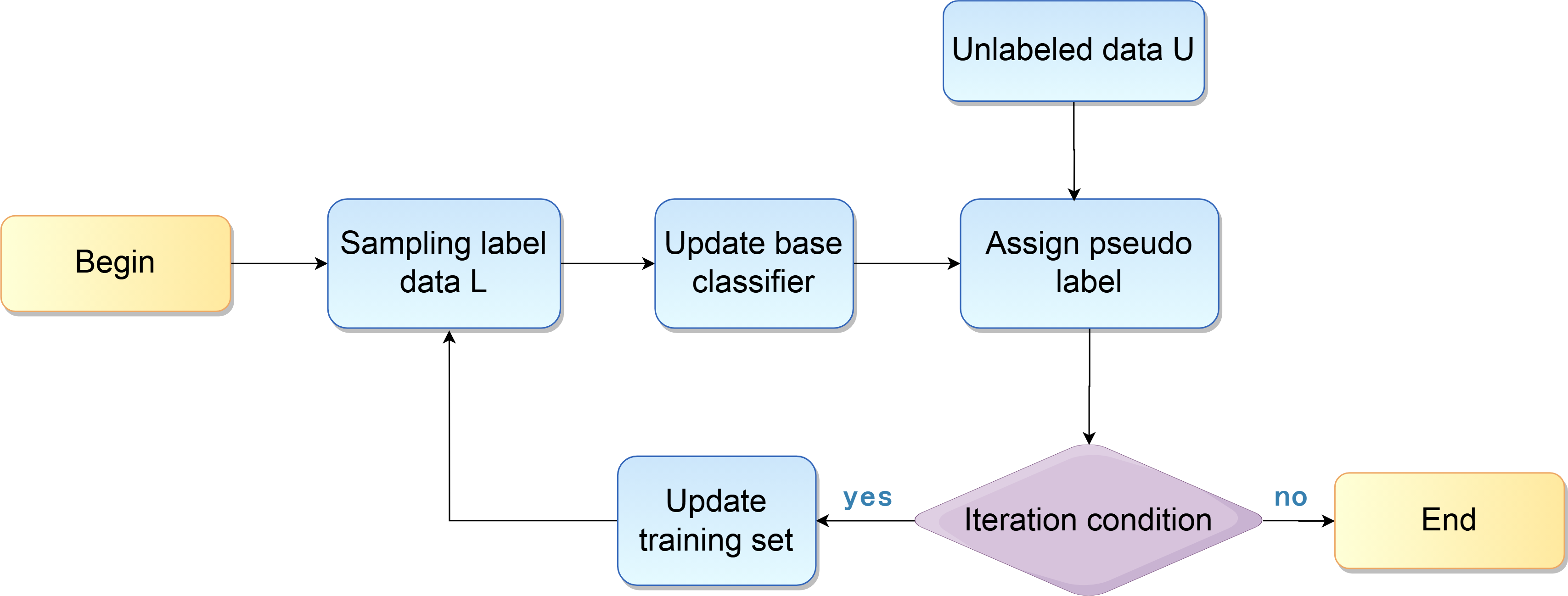

Supplement: S13 Fig — This figure was generated by Figdraw (www.figdraw.com) tool. (TIF) [file pcbi.1014253.s013.tif]
